# Measurable residual mutated *IDH2* before allogeneic transplant for acute myeloid leukemia

**DOI:** 10.1038/s41409-024-02449-2

**Published:** 2024-10-25

**Authors:** Gege Gui, Niveditha Ravindra, Pranay S. Hegde, Georgia Andrew, Devdeep Mukherjee, Zoë Wong, Jeffery J. Auletta, Firas El Chaer, Evan C. Chen, Yi-Bin Chen, Adam Corner, Steven M. Devine, Sunil G. Iyer, Antonio Martin Jimenez Jimenez, Marcos J. G. De Lima, Mark R. Litzow, Partow Kebriaei, Wael Saber, Stephen R. Spellman, Scott L. Zeger, Kristin M. Page, Laura W. Dillon, Christopher S. Hourigan

**Affiliations:** 1 Fralin Biomedical Research Institute, Virginia Tech FBRI Cancer Research Center, Washington, DC USA; 2 Department of Biostatistics, Johns Hopkins Bloomberg School of Public Health, Baltimore, MD USA; 3 Laboratory of Myeloid Malignancies, Hematology Branch, National Heart, Lung, and Blood Institute, National Institutes of Health, Bethesda, MD USA; 4 Center for International Blood and Marrow Transplant Research, NMDP, Minneapolis, MN USA; 5 The Ohio State University College of Medicine, Columbus, OH USA; 6 University of Virginia, Charlottesville, VA USA; 7 Dana-Farber Cancer Institute, Boston, MA USA; 8 Massachusetts General Hospital, Boston, MA USA; 9 Bio-Rad Laboratories, Pleasanton, CA USA; 10 Columbia University Irving Medical Center, New York, NY USA; 11 Sylvester Comprehensive Cancer Center, Miami, FL USA; 12 Mayo Clinic, Rochester, MN USA; 13 The University of Texas MD Anderson Cancer Center, Houston, TX USA; 14 Center for International Blood and Marrow Transplant Research, Medical College of Wisconsin, Milwaukee, WI USA

**Keywords:** Translational research, Clinical genetics, Acute myeloid leukaemia

## Abstract

Routine genetic profiling of acute myeloid leukemia (AML) at initial diagnosis has allowed subgroup specific prognostication, drug development, and clinical management strategies. The optimal approach for treatment response assessment for AML subgroups has not yet however been determined. A nationwide cohort of 257 adult patients in first remission (CR1) from AML associated with an *IDH2* mutation (*IDH2*m) undergoing allogeneic transplant during the period 2013–2019 in the United States had rates of relapse and survival three years after transplantation of 24% and 71%, respectively. Pre-transplant clinical flow cytometry assessment was not useful in stratifying patients based on risk of post-transplant relapse or death. DNA-sequencing was performed on CR1 blood collected within 100 days before transplant. Persistent detection of *IDH2m* was common (51%) and associated with increased relapse and death compared to testing negative. Co-mutation at initial diagnosis with mutated *NPM1* and/or *FLT3*-ITD was common in this cohort (41%) and use of these validated MRD markers provided superior stratification compared to *IDH2m* testing. Patients testing negative for *IDH2*m prior to transplant had low relapse-related death, regardless of conditioning intensity. Post-transplant relapse rates for those with persistently detectable *IDH2m* in pre-transplant remission were lower after the FDA approval of enasidenib in August 2017.

## Introduction

Acute myeloid leukemia (AML) is a type of rare but fatal blood cancer with around 20,000 new cases in the United States per year and an overall survival (OS) of 32% [1]. Mutational sub-classification of AML has created opportunities for specific prognostication, management, and development of targeted therapies [2–4]. Mutations in isocitrate dehydrogenase (*IDH*) genes are commonly observed in patients with AML, with *IDH2* mutations detected at around 10–15% of all patients at AML diagnosis [5, 6]. Two specific *IDH2* mutations, R140 and R172, compose around 80% and 20% of all *IDH2*-mutated cases, respectively [7]. Enasidenib, a targeted inhibitor of *IDH2* mutations, was approved by the United States Food and Drug Administration to treat relapsed or refractory AML in August 2017 and is under study for other indications [8–11]. Allogeneic hematopoietic cell transplant (alloHCT) is an important, potentially curative, therapy for many patients with *IDH2* mutated AML.

Measurable residual disease (MRD) test positivity in patients with AML during first complete remission (CR1) is associated with adverse clinical outcomes after alloHCT [12–23]. While a variety of AML MRD testing methods are possible, a molecular approach is typically preferred when both a validated target and test are available [12, 24–26]. The most common AML-specific somatic mutations, mutated *NPM1* and *FLT3* internal tandem duplication (ITD), are well-validated molecular targets for AML MRD testing [17, 27–31]. While other mutations are commonly detected at initial diagnosis, the prognostic significance of detecting individual, or combinations of, AML-associated mutations in remission have not been fully determined. Selecting and validating additional targets for molecular MRD monitoring will assist relapse prediction and facilitate early intervention, especially for disease subgroups with targeted therapies available [32–37]. While MRD in *IDH*-mutated AML has been studied previously, cohort sample size has limited conclusions [6, 38, 39], and large studies examining the significance of *IDH2* as a potential AML MRD target are of particular interest due to the possibility of targeted clinical intervention.

The Pre-MEASURE project was designed to assess, in a nation-wide retrospective cohort of adults with AML in CR1 prior to first alloHCT, the association of pre-transplant remission blood testing by DNA-sequencing with post-transplant clinical outcomes. We present here the findings associated with persistent *IDH2* mutation (*IDH2*m) detection.

## Materials and methods

### Study cohort

Patients 18 years or older with AML reported as associated with an *IDH2m* at diagnosis, who achieved CR1 and underwent alloHCT at one of 112 sites from the Center for International Blood and Marrow Transplant Research (CIBMTR) from 2013–2019, registered to participate in the CIBMTR database (NCT01166009) and repository (NCT04920474) protocols, and with a remission blood sample collected within 100 days of alloHCT were included in the study (median: 8.5 days, range 0–71). All patients gave written informed consent in accordance with the Declaration of Helsinki. Clinical outcome data were provided by CIBMTR including time to death or censoring, time to relapse, and graft-versus-host disease (GvHD, acute or chronic) when available. Other baseline characteristics were also extracted from the database including age, sex, race, ethnicity, hematopoietic cell transplant specific comorbidity (HCT-CI), Karnofsky performance status (KPS), secondary AML, European LeukemiaNet (ELN) 2017 risk group, baseline *IDH2* mutational status, conditioning regimen, graft type, donor group, anti-thymocyte globulin (ATG) usage, site reported flow cytometry MRD status, and month/year transplanted.

### Residual variant detection by next generation sequencing

Genomic DNA was extracted from remission blood obtained from the CIBMTR repository and sequenced as described previously [17]. In short, ultrasensitive error-corrected next generation sequencing (NGS) targeting mutational hotspot regions of the *IDH2*, *NPM1*, and *FLT3* genes was performed utilizing an automated workflow with pre- and post-PCR separation. The assay was validated to detect *IDH2* variants down to at least 0.1% variant allele fraction (VAF) by performing serial dilutions of AML patient DNA containing known *IDH2* R140Q, R172K, or R132H mutations into normal DNA at a range of 5% to 0.005%. *NPM1* and *FLT3*-ITD variants were called down to 0.01% VAF. NGS libraries were sequencing on a NovaSeq 6000 (Illumina) with unique dual indices. Bioinformatic pipelines were used to perform error-corrected variant calling followed by filtering to identify residual disease. Raw FASTQ files are available at the NCBI Sequence Read Archive (SRA) (Accession: PRJNA834073 and PRJNA1051602).

### Digital droplet PCR (ddPCR)

A subset of 127 *IDH2* variants identified by NGS were orthogonally validated using ddPCR on the Bio-Rad QX200 or QX600 system as described previously [17].

### Statistical analysis

The primary outcomes were OS and cumulative incidence of relapse with non-relapse related mortality (NRM) as a competing risk. The day of transplant was considered as time 0, and the median follow-up time was calculated for censored patients. Kaplan–Meier (KM) estimation and log rank tests were used to calculate OS and relapse-free survival endpoints. Cox proportional hazards models were fitted, with forward selection by analysis of variance or Lasso penalty for variable selection, and the proportional hazards assumptions were validated. Fine-Gray regression models were used to examine the cumulative incidence of relapse with NRM as a competing risk, and Bayesian information criterion (BIC) was used for model selection. Potential interactions between NGS *IDH2* MRD status and clinical characteristics were tested. KM curves were visualized up to 3 years based on the number of patients at risk, but the p-values included were obtained using full data. Two-sided p-values less than or equal 0.05 were considered as statistically significant. R version 4.3.0 was used to perform statistical analysis, generate figures and tables.

## Results

### Clinical characteristics and post-transplant clinical outcomes

A total of 257 adults with *IDH2*-mutated AML undergoing alloHCT in CR1 at a CIBMTR reporting site between 2013 and 2019 were included in this study (Table 1). The median age was 60.1 (range 19.8–79.3), 84% were Caucasian, 50% were female, 57% had a Karnofsky performance score of 90 or above, and 48% had an HCT-CI comorbidity index of 3 or greater. In addition to *IDH2* mutations in all patients, *NPM1* and/or *FLT3*-ITD mutations were reported at initial diagnosis in 41%. Transplants were from peripheral blood 75% of the time, 49%  had myeloablative conditioning, and 60% were from matched unrelated donors.

**Table 1:** Patient clinical characteristics.

Variable	Count (%)	Variable	Count (%)
**Age (years)**		**HCT-comorbidity index**	
<60	127 (49%)	0	53 (21%)
≥60	130 (51%)	1,2	80 (31%)
**Sex**		>2	124 (48%)
Female	128 (50%)	**Karnofsky Score**	
Male	129 (50%)	<90	106 (41%)
**Conditioning Intensity**		≥90	147 (57%)
MAC	127 (49%)	**ATG Usage**	
RIC with melphalan	62 (24%)	No	202 (79%)
RIC without melphalan	46 (18%)	Yes	55 (21%)
NMA	22 (9%)	**Graft Type**	
**Race**		Bone Marrow	39 (15%)
Caucasian	217 (84%)	Cord Blood	24 (9%)
Other	32 (12%)	Peripheral Blood	194 (75%)
**Donor Group**		**ELN Risk Group**	
Cord Blood	18 (7%)	Favorable	44 (17%)
Haploidentical Related	20 (8%)	Intermediate	111 (43%)
HLA-identical Sibling	37 (14%)	Adverse	102 (40%)
Matched Unrelated	155 (60%)	**AML Group**	
Mismatched	21 (8%)	De novo	226 (88%)
Multiple Donors	6 (2%)	Therapy-Related	10 (4%)
**Site-reported Flow Cytometry MRD**		Transformed MDS/MPN	21 (8%)
Negative	222 (86%)	**Baseline Mutation**	
Positive	25 (10%)	*NPM1* and/or *FLT3*-ITD	106 (41%)

*MAC* myeloablative conditioning, *RIC* reduced intensity conditioning, *NMA* nonmyeloablative, *AML* acute myeloid leukemia, *HLA* human leukocyte antigen, *MRD* measurable residual disease, *HCT* hematopoietic cell transplantation, *ATG* antithymocyte globulin, *ELN* European LeukemiaNet, *MDS* myelodysplastic syndrome, *MPN* myeloproliferative neoplasm, *ITD* internal tandem duplication.

**Fig. 1:**
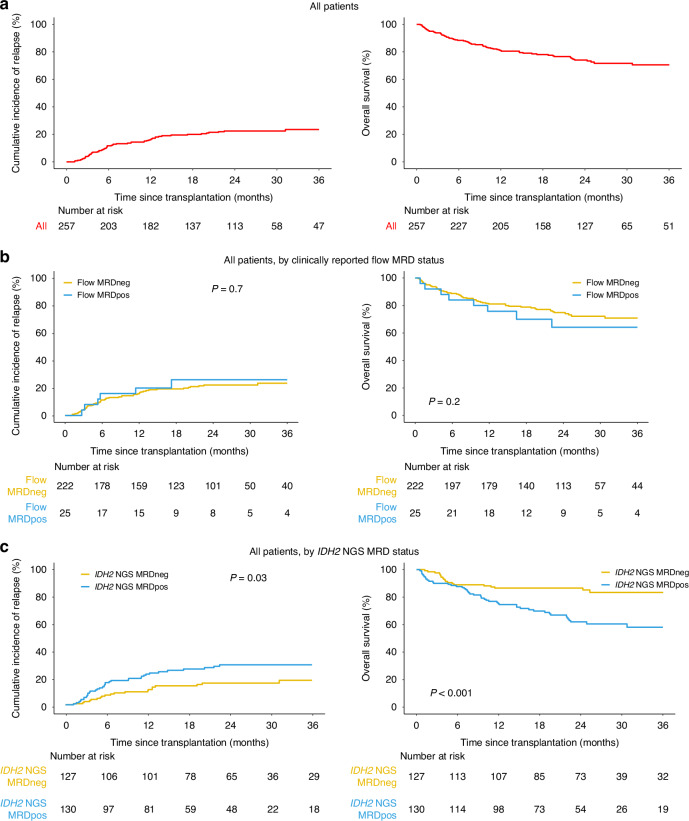
Clinical outcomes of AML patients with *IDH2* mutations at baseline and the association with MRD after allogeneic hematopoietic cell transplant. Cumulative incidence of relapse (left) and overall survival (right) are shown for *IDH2* mutated patients (**a**) for the entire cohort (**b**) based on the presence (Flow MRDpos) or absence (Flow MRDneg) of reported clinical flow cytometry measurable residual disease (MRD), and (**c**) based on the presence (*IDH2* NGS MRDpos) or absence (*IDH2* NGS MRD neg) of residual *IDH2* variants by next generation sequencing (NGS) MRD assay.

Overall, the 1-year and 3-year cumulative incidence of relapse in this cohort was estimated as 16% and 24%, respectively (Fig. 1a, Supplementary Fig. 1). Of 57 relapses recorded, 41 (72%) occurred within 12 months following transplantation. For all baseline characteristics studied (Table 1), using univariable competing risk regression considering NRM as the competing risk event, only cord blood compared to peripheral blood as a graft type (HR: 2.3, 95% CI 1.2–4.5; Supplementary Fig. 2A) was related with higher relapse risk.

Overall, the 1-year and 3-year OS in this cohort were estimated as 81% and 71%, respectively (Fig. 1a). Median follow-up time among censored patients was 25 months. Two baseline characteristics identified by univariable Cox regression analysis as associated with differences in OS were HCT-CI (3+ vs. 0, HR: 2.8, 95% CI: 1.3–5.9) and AML group (transformed from myelodysplastic syndrome/myeloproliferative neoplasm vs. de novo HR: 2.1, 95% CI: 1.1–4.3; Supplementary Fig. 2B).

### Pre-transplant flow cytometry and residual *IDH2m* detection

Flow cytometry is commonly performed for patients with AML in remission prior to transplant as a test of MRD to estimate post-transplant relapse risk [12], although the clinical utility of such testing as currently performed has been questioned [17, 40]. 247 of the 257 (96%) patients in this cohort had pre-transplant remission flow cytometry results reported to the CIBMTR registry, of which 25 (10%) tested positive. Testing positive by flow cytometry pre-transplant did not predict any differences in relapse or OS compared with those testing negative (Fig. 1a).

Testing for *IDH2m* persistence for patients with AML in remission prior to transplant is not routinely performed clinically. A custom error-corrected NGS assay was validated to detect *IDH2* mutations down to a variant allele fraction (VAF) of at least 0.1% (Supplementary Fig. 3A). NGS analysis detected residual *IDH2m* in CR1 pre-transplant blood samples from 130 patients (51%) with VAFs ranging from 0.05 to 56% (median: 3%; Supplementary Fig. 3, Supplementary Table 1). Those with *IDH2m* detected (*IDH2* NGS MRDpos) had increased relapse (3 yrs: 29% vs. 18%, +11%, 95% CI: 0.2% to 22%; overall *p* = 0.03), decreased OS (3 yrs: 58% vs. 83%, −25%, 95% CI: −13% to −38%; overall *p* < 0.001), and decreased relapse-free survival (RFS 3 yrs: 53% vs. 70%, −17%, 95% CI: −4% to −30%; overall *p* = 0.002) compared to those testing negative (*IDH2* NGS MRDneg, Fig. 1c). The OS for *IDH2* NGS MRDneg patients was estimated to be 83% (95% CI: 75% to 89%, *p* < 0.001) at 3 years and only 6 of the 21 deaths in this group were relapse related.

### Association of age, residual *IDH2m* burden and type with clinical outcome

**Fig. 2:**
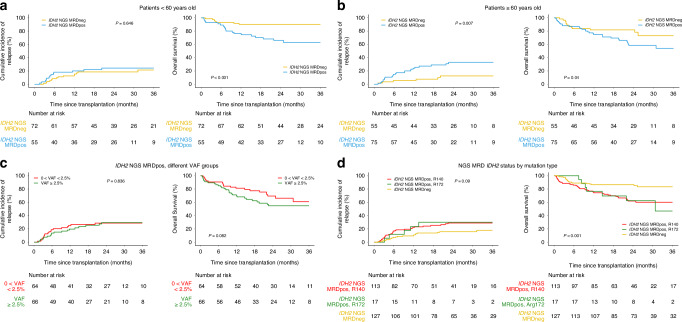
Baseline characteristics for *IDH2* mutated AML patients and the association with clinical outcomes after allogeneic hematopoietic cell transplant. Cumulative incidence of relapse (left) and overall survival (right) are shown for *IDH2* mutated patients by the presence (*IDH2* NGS MRDpos) or absence (*IDH2* NGS MRDneg) of residual *IDH2* variants and by (**a**) age group below 60 years old, (**b**) age group 60 years or above, (**c**) different variant allele fraction (VAF) groups, and (**d**) by *IDH2* mutation type.

To study the association of *IDH2m* detection in pre-transplant CR1 and clinical outcomes in younger and older patients, subgroups were created by using 60 years of age as a cutoff (≥60 vs. <60). For both age groups, decreased OS was observed for those testing positive for *IDH2m* compared to those testing negative (<60 yrs, 3 yrs: 62% vs. 90%, −28%, 95% CI: −12% to −43%, overall *p* < 0.001; ≥60 yrs, 3 yrs: 54% vs. 73%, −19%, 95% CI: −1% to −40%, overall *p* = 0.04; Fig. 2a, b; Supplementary Fig. 4). OS for the younger group testing negative for persistent *IDH2m* was 90% (95% CI: 81% to 95%, *p* < 0.001); among 9 patients who died in this group only 3 experienced relapse. Older patients had an increased rate of relapse when testing positive for persistent *IDH2m* compared to those testing negative (3 yrs: 33% vs. 12%, +21%, 95% CI: 6% to 36%, overall *p* = 0.007). Interestingly, the level of *IDH2m* persistence was significantly higher in older versus younger patients (median VAFs: 5.4% vs. 1.4%, ≥60 yrs vs. <60 yrs, *p* = 0.01). To examine a potential dose effect of *IDH2m* persistence, patients were divided into two VAF subgroups with 2.5% as the cutoff, but the risk of relapse was not significantly different between the VAF groups (Fig. 2c). Among patients with residual *IDH2m* detected, 13% (*n* = 17) had R172 variants and the remaining had R140 variants. The type of residual *IDH2m* did not further stratify the risks of clinical outcomes (Fig. 2d).

### Co-occurrence of mutated *IDH2* with mutated *NPM1* and *FLT3*-ITD

**Fig. 3:**
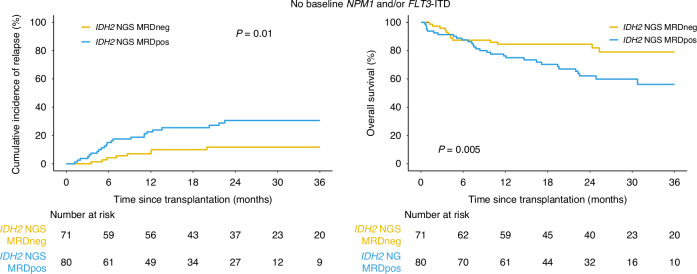
Clinical outcomes for *IDH2*-mutated AML patients without baseline mutations in *NPM1* or *FLT3*-ITD and the association with residual *IDH2* variants. Cumulative incidence of relapse on the left and overall survival on the right for patients based on the presence (*IDH2* NGS MRDpos) or absence (*IDH2* NGS MRDneg) of residual *IDH2* variants.

As *IDH2* mutated AML often also has mutations in *NPM1* and/or *FLT3*-ITD [2, 4], the cohort was separated into those with (*n* = 106, 41%) or without (*n* = 151, 59%) either of these two mutations reported at initial diagnosis. In the *IDH2* mutated patients who did not also have mutated *NPM1* and/or *FLT3*-ITD at baseline, *IDH2m* were commonly detected in pre-transplant remission blood (*n* = 80, 53%) and was associated with increased relapse and decreased OS compared with testing negative (relapse 3 yrs: 31% vs. 12%, +19%, 95% CI: 6% to 32%, overall *p* = 0.01; OS 3 yrs: 56% vs. 79%, −23%, 95% CI: −6% to −40%, overall *p* = 0.005; Fig. 3, Supplementary Fig. 5).

**Fig. 4:**
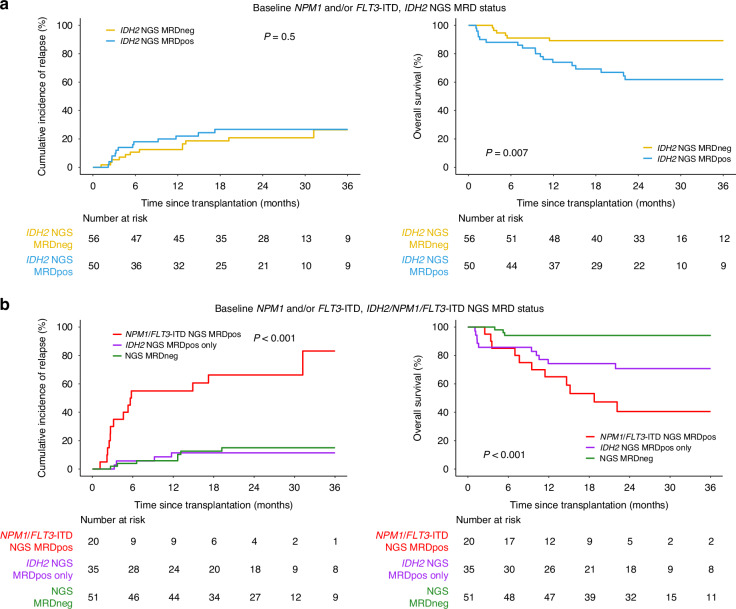
Clinical outcomes of *IDH2*-mutated AML patients with co-mutated *NPM1* or *FLT3*-ITD at baseline and the association with residual variants. Cumulative incidence of relapse on the left and overall survival on the right for patients based on (**a**) the presence (*IDH2* NGS MRDpos) or absence (*IDH2* NGS MRDneg) of residual *IDH2* variants or (**b**) the presence of residual *NPM1* and/or *FLT3-*ITD variants (*NPM1*/*FLT3*-ITD NGS MRDpos), the presence of only residual *IDH2* variants (*IDH2* NGS MRDpos), or the absence of residual variants (NGS MRDneg).

In contrast, for patients known to also have mutations in *NPM1* and/or *FLT3*-ITD at initial diagnosis (*n* = 106), detection of persistent *IDH2m* pre-transplant was also common (*N* = 50, 47%) but was prognostic only for OS and not relapse (Fig. 4a, Supplementary Fig. 6). Since the persistence of *NPM1* and/or *FLT3*-ITD variants in pre-transplant CR1 blood has already been shown to be strongly associated with worse post-transplant clinical outcomes [17], the utility of these markers was validated in this specific setting. As anticipated the detection of persistent *NPM1* and/or *FLT3*-ITD variants (*n* = 20, 19%) was strongly associated with increased relapse, while those with only residual *IDH2m* detected (*n* = 35, 33%) had similar relapse risk as those testing negative (3 yrs: 65% vs. 12% vs. 16%; overall *p* < 0.001; *p* < 0.001 for *NPM1*/*FLT3*-ITD vs. negative, *p* = 0.8 for *IDH2m* only vs. negative; Fig. 4b). Both residual mutation positive groups had inferior OS compared to the negative group while patients with residual *NPM1*/*FLT3*-ITD had the worst estimated OS (3 yrs: 41% vs. 71% vs. 94%; overall *p* < 0.001). None of the patients testing negative (*n* = 51, 48%) experienced relapse-related mortality among the 4 reported death events.

### Modifying effects of other clinical characteristics on residual *IDH2*m detection

**Fig. 5:**
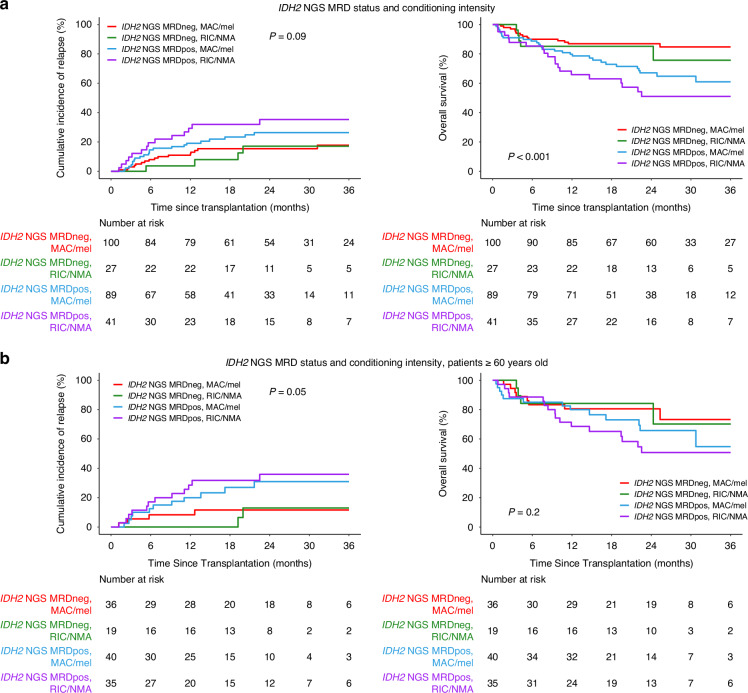
Evaluation of conditioning intensity modifying the association of residual *IDH2* mutations and clinical outcomes. Cumulative incidence of relapse on the left and overall survival on the right for (**a**) the entire cohort of *IDH2*-mutated AML patients, and (**b**) those ≥60 years of age based on the presence (*IDH2* NGS MRDpos) or absence (*IDH2* NGS MRDneg) of residual *IDH2* variants and conditioning intensity received. MAC myeloablative conditioning, Mel melphalan, RIC reduced intensity conditioning, NMA nonmyeloablative conditioning.

Given evidence that the intensity of the conditioning regimen used prior to transplant can impact clinical outcomes in MRD positive patients [22], we next examined the modifying effect of conditioning intensity and *IDH2m* persistence. Patients were grouped into low intensity (reduced intensity conditioning (RIC) and nonmyeloablative (NMA)) and high intensity (RIC with melphalan and myeloablative conditioning (MAC)) regimens. When considering all patients, those with *IDH2m* persistence who received RIC/NMA had the highest rate of relapse (3 yrs: 35%, 95% CI: 20–51%) and lowest OS (3 yrs: 51%, 95% CI: 34–66%; Fig. 5a, Supplementary Fig. 7), but there was no statistically significant difference among the residual *IDH2*m patients based on treatment regimen. Conditioning intensity was not randomized in this retrospective observational study but was likely determined, at least in part, by judgements regarding patient performance status and comorbidity. Age is an important factor, and as expected RIC/NMA was more frequently given to older patients (<60 yrs vs. ≥60 yrs: 11% vs. 42%, Chi-squared test *p* < 0.001). While there were few cases of patients <60 years old with persistent *IDH2m* who received RIC/NMA, when looking at those ≥60 years, higher rates of relapse were observed in patients testing *IDH2m* positive when given RIC/NMA compared to those testing negative (1 yr: 29% vs. 0, +29%, 95% CI: +13.3% to +43.8%, *p* < 0.001; Fig. 5b).

For the full cohort, multivariable Cox regression analysis for OS indicated that patients with persistent *IDH2*m had a higher risk of relapse compared to those testing negative (HR: 2.5, 95% CI: 1.5–4.2, *p* < 0.001) after adjusting for other variables of interest, which also had significant differences in OS risk including hematopoietic cell transplantation-comorbidity index and age group (Table 2). Residual *IDH2m* remained prognostic in relapse using competing risk regression (*IDH*2 NGS MRDpos vs. *IDH*2 NGS MRDneg, HR: 2.1, 95% CI: 1.4–5.7, *p* = 0.003) when considering graft type and *IDH1* baseline status.

**Table 2:** Estimated hazard ratios of clinical outcomes in multivariable analyses.

Overall Survival: full cohort			Relapse: full cohort		
	Hazard ratio (95% CI)	*P*		Hazard ratio (95% CI)	*P*
NGS MRD: *IDH2* Positive	2.5 (1.5–4.2)	<0.001	Graft Type: Cord Blood	2.9 (1.4–5.7)	0.003
HCT-CI: 1,2	1.5 (0.6–3.4)	0.4	Graft Type: Bone Marrow	1.8 (0.9–3.5)	0.08
HCT-CI: >2	3.1 (1.5–6.7)	0.003	NGS MRD: *IDH2* Positive	2.1 (1.3–3.7)	0.005
Age: ≥60	1.7 (1.1–2.8)	0.03	*IDH1* Baseline: Positive	2.8 (1.3–5.8)	0.007

Overall survival: no baseline *NPM1*/*FLT3*-ITD			Relapse: no baseline *NPM1*/*FLT3*-ITD		
	Hazard ratio (95% CI)	*P*		Hazard ratio (95% CI)	*P*
NGS MRD: *IDH2* Positive	2.5 (1.3–4.7)	0.006	Graft Type: Cord Blood	5.8 (2.3–14.9)	<0.001
			Graft Type: Bone Marrow	2.3 (1.0–5.6)	0.06
			NGS MRD: *IDH2* Positive	3.3 (1.5–7.2)	0.003
			*IDH1* Baseline: Positive	4.3 (1.5–12.5)	0.008

Overall survival: with baseline *NPM1*/*FLT3*-ITD			Relapse: with baseline *NPM1*/*FLT3*-ITD		
	Hazard ratio (95% CI)	*P*		Hazard ratio (95% CI)	*P*
NGS MRD: *NPM1/FLT3-*ITD Positive	10 (3.2–31)	<0.001	NGS MRD: *NPM1/FLT3-*ITD Positive	21.0 (7.0–63)	<0.001
			Age: ≥60	5.0 (1.4–18)	0.015
NGS MRD: *IDH2* Positive, *NPM1/FLT3*-ITD Negative	4.4 (1.4–13.7)	0.012	Age: every 1 year above 40	0.95 (0.92–0.97)	<0.001
			*IDH1* Baseline: Positive	4.7 (1.5–14.3)	0.007
			ATG usage: yes	2.9 (1.1–7.3)	0.026
			Sex: male	0.3 (0.1–0.9)	0.027

*NGS* next generation sequencing, *MRD* measurable residual disease, *HCT-CI* hematopoietic cell transplantation-comorbidity index, *ATG* antithymocyte globulin, *CI* confidence interval.

The prognostic significance for persistent *IDH2m* remained when patients did not have *NPM1* and/or *FLT3*-ITD mutations at baseline (HR for OS: 2.5, 95% CI: 1.3–4.7; HR for relapse: 3.3, 95% CI: 1.5–7.2; Table 2) when including other clinically important characteristics in the regression model. For patients with *NPM1* and/or *FLT3*-ITD mutations at baseline, residual *NPM1* and/or *FLT3*-ITD variants was the most significant variable in predicting both OS and relapse outcomes (OS, HR: 10, 95% CI: 3.2–31.2, *p* < 0.001; relapse, HR: 21, 95% CI: 7–63, *p* < 0.001), while residual *IDH2*m was only significant for relapse (HR: 4.36, 95% CI: 1.4–13.7, *p* = 0.01), when adjusting for other covariates of interest (Table 2).

**Fig. 6:**
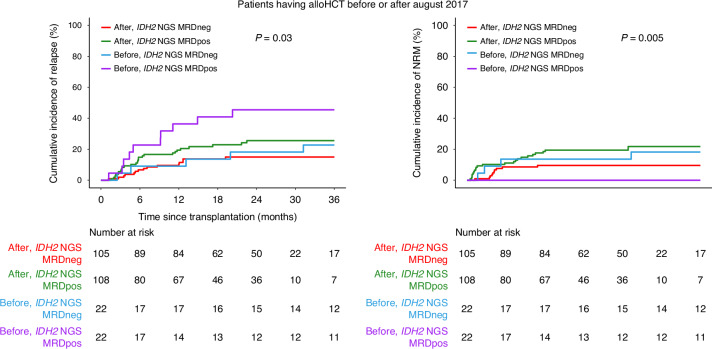
The association of enasidenib drug approval date and residual *IDH2* mutations on clinical outcomes. Cumulative incidence of relapse on the left and non-relapse related mortality (NRM) on the right for *IDH2*-mutated AML patients based on the date of transplant occurring before or after the approval date of enasidenib (August 2017) and the presence (*IDH2* NGS MRDpos) or absence (*IDH2* NGS MRDneg) of residual *IDH2* variants.

Since the *IDH2* inhibitor enasidenib was approved by the FDA on August 1st of 2017, patients were divided into two subgroups with alloHCT date before (*n* = 44, 17%) or after (*n* = 213, 83%) the date of drug approval to examine the potential therapeutic impact. Although the patient numbers were not balanced between the two groups, the difference in rates of relapse between the residual *IDH2*m positive and negative groups was greater before the approval date versus after (before: 45% vs. 23%, after: 26% vs. 15%, overall *p* = 0.03; Fig. 6, Supplementary Fig. 8A). There was also a difference in NRM comparing those testing positive versus negative for residual *IDH2m* (before: 0 vs. 18%, after: 22% vs. 10%, overall *p* = 0.005), with 9/10 death events relapse related for patients in the *IDH2*m positive group transplanted before August 2017. The same patterns in clinical outcomes were observed when removing patients with baseline *FLT3*-ITD mutation (Supplementary Fig. 8B).

## Discussion

Presented here is the largest observational study of patients with *IDH2*-mutated AML examining the association between the detection of residual *IDH2*m in the blood during CR1 prior to the first alloHCT and post-transplant clinical outcomes. Around half of the patients examined had residual *IDH2* mutations detected in the blood during CR1 using ultra-deep error-corrected NGS, which was associated with statistically significant increased rates of relapse and decreased OS. Patients without detectable persistence of *IDH2m* generally had very high survival after transplantation. Further exploratory subgroup and multivariable analyses showed interesting variations in clinical outcomes for patients given different conditioning intensity regimens and with various baseline or transplant-related characteristics, although are limited by subgroup cohort size, imbalance in age groups, and non-random conditioning intensity assignment. In addition, further study on the impact of additional baseline mutational characteristics and targeted inhibitor use was not possible in this large retrospective registry study.

Mutated *NPM1* and *FLT3*-ITD are now well-validated AML MRD targets [17, 27–31], and this *IDH2*-mutated cohort was further studied by dividing into two subgroups based on whether they also had either of these two mutations reported at initial diagnosis. In patients with *IDH2*-mutated AML also co-mutated with either *NPM1* and/or *FLT3*-ITD, the persistence of only *IDH2m* pre-transplant was not associated with increased relapse post-transplant. Persistence of *NPM1* and/or *FLT3*-ITD variants was however very strongly associated with increased relapse and increased death after transplant and proves to be a superior marker to *IDH2m* in these patients.

In those patients diagnosed with *IDH2m* AML that was not co-mutated with either *NPM1* and/or *FLT3*-ITD, persistent detection of *IDH2*m pre-transplant was common and associated with increased relapse and death after transplant.

The prognostic significance of persistent *IDH2m* pre-transplant was superior to reported clinical flow cytometry results but not as great as that reported in different patient subsets for other molecular AML MRD markers. Definitions based on patient age, VAF, or mutation type did not improve stratification for post-transplant relapse or survival outcomes.

While the primary intent of this study was to determine the ability of pre-transplant *IDH2m* MRD testing to predict post-transplant relapse, we observed in multiple analyses a trend for the persistence of *IDH2m* pre-transplant having a greater than expected effect on post-transplant survival relative to relapse rates. There are many potential explanations for this finding, ranging from differences in disease biology such that relapses from those with detectable pre-transplant disease were more likely to be fatal, to differences in pre- and post-transplant therapy (including off-label use of *IDH2* inhibitor therapy), to the possibility that NRM is higher in those with persistent *IDH2* mutated cells due to differences in immune status [41]. This hypothesis-generating observation is worthy of further study but is beyond the scope of this project.

In summary, persistence of *IDH2* variants in the blood during first remission prior to first alloHCT is common and is associated with increased relapse and death post-transplant. Patients with AML co-mutated with either *NPM1* or *FLT3*-ITD should be tested for MRD using mutated *NPM1* or *FLT3*-ITD rather than *IDH2* persistence.

## Supplementary information

Supplementary Material

## Data Availability

Sequencing data are available at the NCBI Sequence Read Archive (SRA) (Accession: PRJNA834073 and PRJNA1051602). Clinical data will be published by CIBMTR as a resource.
