# Supplementary material for: Measurable residual mutated IDH2 before allogeneic transplant for acute myeloid leukemia
Source: Bone Marrow Transplant. 2024 Oct 25;60(2):144–53. doi: 10.1038/s41409-024-02449-2 (PMC11810785; doi:10.1038/s41409-024-02449-2)
Supplement: Supplementary file 1 — Supplementary Material [file 41409_2024_2449_MOESM1_ESM.pdf]

Supplementary Data

Measurable Residual Mutated *IDH2* before Allogeneic Transplant for Acute Myeloid Leukemia

Gege Gui[1,2], Niveditha Ravindra[3], Pranay S. Hegde[3], Georgia Andrew[3], Devdeep Mukherjee[3], Zoë Wong[3], Jeffery J. Auletta[4,5], Firas El Chaer[6], Evan C. Chen[7], Yi-Bin Chen[8], Adam Corner[9], Steven M. Devine[4], Sunil G. Iyer[10], Antonio Martin Jimenez Jimenez[11], Marcos J.G. De Lima[5], Mark R. Litzow[12], Partow Kebriaei[13], Wael Saber[14], Stephen R. Spellman[4], Scott L. Zeger[2], Kristin M. Page[14], Laura W. Dillon[3]*, Christopher S. Hourigan[1]*
*LWD and CSH contributed equally to this work

[1]Fralin Biomedical Research Institute, Virginia Tech FBRI Cancer Research Center, Washington, DC
[2]Department of Biostatistics, Johns Hopkins Bloomberg School of Public Health, Baltimore, MD
[3]Laboratory of Myeloid Malignancies, Hematology Branch, National Heart, Lung, and Blood Institute, National Institutes of Health, Bethesda, MD
[4]Center for International Blood and Marrow Transplant Research, NMDP, Minneapolis, MN
[5]The Ohio State University College of Medicine, Columbus, OH
[6]University of Virginia, Charlottesville, VA
[7]Dana-Farber Cancer Institute, Boston, MA
[8]Massachusetts General Hospital, Boston, MA
[9]Bio-Rad Laboratories, Pleasanton, CA
[10]Columbia University Irving Medical Center, New York, NY
[11]Sylvester Comprehensive Cancer Center, Miami, FL
[12]Mayo Clinic, Rochester, MN
[13]The University of Texas MD Anderson Cancer Center, Houston, TX
[14]Center for International Blood and Marrow Transplant Research, Medical College of Wisconsin, Milwaukee, WI

Table of Contents

Supplementary Figure 1. Clinical outcomes of AML patients with *IDH2* mutations at baseline and the association with MRD after allogeneic hematopoietic cell transplant.	Page 2
Supplementary Figure 2. Univariable Cox regression for relapse and overall survival.	Page 3
Supplementary Figure 3. Detection of residual variants in pretransplant blood of *IDH2* mutated AML patients during complete remission.	Page 4
Supplementary Figure 4. NGS MRD status for *IDH2*-mutated AML patients and the association with clinical outcomes after allogeneic hematopoietic cell transplant stratified by age, variant allele fraction (VAF), and mutation type.	Page 5
Supplementary Figure 5. NGS MRD status for *IDH2*-mutated AML patients without *NPM1*/*FLT3*-ITD mutations at baseline and the association with clinical outcomes after allogeneic hematopoietic cell transplant stratified by age group.	Page 6
Supplementary Figure 6. NGS MRD status for *IDH2*-mutated AML patients with *NPM1*/*FLT3*-ITD mutations at baseline and the association with clinical outcomes after allogeneic hematopoietic cell transplant.	Page 7
Supplementary Figure 7. NGS MRD status for *IDH2*-mutated AML patients and the association with clinical outcomes after allogeneic hematopoietic cell transplant stratified by conditioning intensity and age group.	Page 8
Supplementary Figure 8. Patient transplant years for *IDH2*-mutated AML patients and the association with clinical outcomes after allogeneic hematopoietic cell transplant stratified by NGS MRD status and baseline mutation.	Page 9
Supplementary Table 1. Variants detected by next-generation sequencing in the blood of *IDH2* mutated AML patients prior to transplant conditioning.	Page 10

Supplementary Figure 1. Clinical outcomes of AML patients with *IDH2* mutations at baseline and the association with MRD after allogeneic hematopoietic cell transplant. Cumulative incidence of non-relapse mortality (NRM, top left) and relapse (top right), relapse-free survival (RFS, bottom left) and overall survival (OS, bottom right) shown at 36 months **(A)** for the entire cohort selected, **(B)** based on the presence (Flow MRD positive) or absence (Flow MRD negative) of reported clinical flow cytometry MRD, and **(C)** based on the presence based on the presence (NGS MRD *IDH2*+) or absence (NGS MRD *IDH2*-) of residual *IDH2* variants by next generation sequencing (NGS) measurable residual disease (MRD) assay. Point estimates at different time points are shown in the table (far right). Overall P values: Gray's test for non-relapse mortality (NRM) and relapse; log-rank test for relapse-free survival (RFS) and overall survival (OS). P values for pointwise estimations at different time points: z-test. Confidence interval, CI; Probability, prob; Month, mo; Year, yr.

A

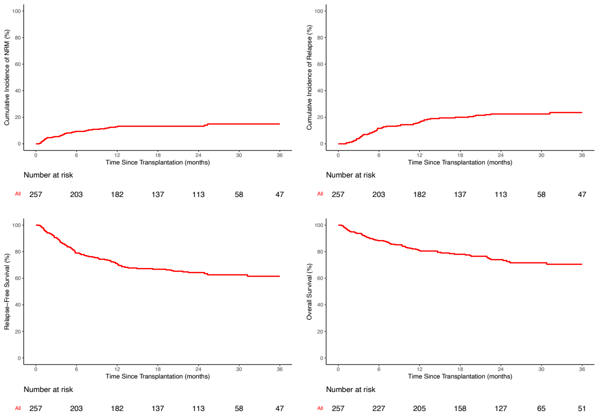

Outcomes	Prob (95% CI)
NRM	n=257
3 mo	5(3-8)%
6 mo	9(6-13)%
1 yr	13(9-17)%
2 yr	13(9-17)%
3 yr	15(10-20)%
Relapse	n=257
3 mo	4(2-6)%
6 mo	12(8-16)%
1 yr	16(11-20)%
2 yr	22(17-28)%
3 yr	24(18-29)%
RFS	n=257
3 mo	91(86-94)%
6 mo	79(73-83)%
1 yr	71(65-76)%
2 yr	64(58-70)%
3 yr	61(55-68)%
OS	n=257
3 mo	94(90-96)%
6 mo	88(84-92)%
1 yr	81(76-85)%
2 yr	74(68-79)%
3 yr	71(64-76)%

B

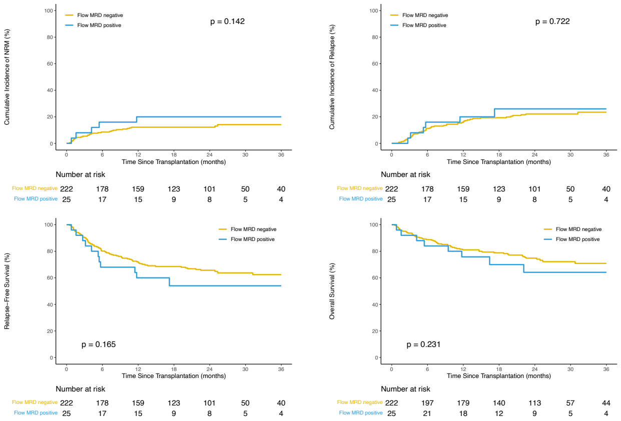

Site-Reported Flow MRD Status

Outcomes	Prob (95% CI)	Prob (95% CI)	p-value
NRM	Flow MRD negative n=222	Flow MRD positive n=25	0.142
3 mo	5(2-8)%	8(0-19)%	0.652
6 mo	9(5-12)%	16(1-31)%	0.336
1 yr	12(8-16)%	20(4-36)%	0.356
2 yr	12(8-16)%	20(4-36)%	0.356
3 yr	14(9-19)%	20(4-36)%	0.494
Relapse	Flow MRD negative n=222	Flow MRD positive n=25	0.722
3 mo	4(1-7)%	4(0-12)%	0.99
6 mo	11(7-15)%	16(1-31)%	0.544
1 yr	16(11-21)%	20(4-36)%	0.622
2 yr	22(17-28)%	26(7-45)%	0.705
3 yr	23(17-30)%	26(7-45)%	0.803
RFS	Flow MRD negative n=222	Flow MRD positive n=25	0.165
3 mo	91(86-94)%	88(67-96)%	0.708
6 mo	80(74-85)%	68(46-83)%	0.209
1 yr	72(66-77)%	60(38-76)%	0.239
2 yr	66(59-72)%	54(32-72)%	0.288
3 yr	62(55-69)%	54(32-72)%	0.448
OS	Flow MRD negative n=222	Flow MRD positive n=25	0.231
3 mo	94(90-96)%	92(72-98)%	0.765
6 mo	89(84-92)%	84(63-94)%	0.535
1 yr	81(75-86)%	76(54-88)%	0.557
2 yr	75(68-80)%	64(40-81)%	0.329
3 yr	71(63-77)%	64(40-81)%	0.544

C

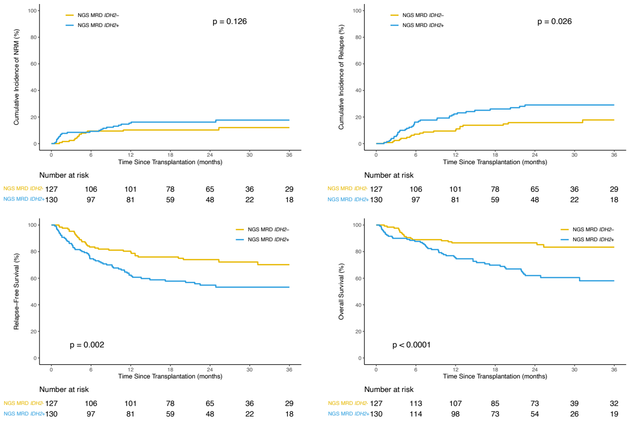

NGS MRD *IDH2* Status

Outcomes	Prob (95% CI)	Prob (95% CI)	p-value
NRM	NGS MRD *IDH2*- n=127	NGS MRD *IDH2*+ n=130	0.126
3 mo	2(0-5)%	8(4-13)%	0.029
6 mo	9(4-15)%	9(4-14)%	0.952
1 yr	10(5-16)%	15(9-22)%	0.217
2 yr	10(5-16)%	16(10-23)%	0.16
3 yr	12(6-18)%	18(11-25)%	0.237
Relapse	NGS MRD *IDH2*- n=127	NGS MRD *IDH2*+ n=130	0.026
3 mo	2(0-5)%	5(1-9)%	0.209
6 mo	7(3-12)%	16(10-23)%	0.022
1 yr	9(4-15)%	22(15-29)%	0.004
2 yr	16(9-22)%	29(21-37)%	0.013
3 yr	18(10-25)%	29(21-37)%	0.047
RFS	NGS MRD *IDH2*- n=127	NGS MRD *IDH2*+ n=130	0.002
3 mo	95(90-98)%	86(79-91)%	0.011
6 mo	83(76-89)%	75(66-81)%	0.079
1 yr	80(72-86)%	62(53-70)%	0.001
2 yr	74(65-81)%	55(46-63)%	0.001
3 yr	70(60-78)%	53(44-62)%	0.01
OS	NGS MRD *IDH2*- n=127	NGS MRD *IDH2*+ n=130	<0.001
3 mo	98(93-99)%	90(83-94)%	0.01
6 mo	89(82-93)%	88(81-92)%	0.748
1 yr	87(79-91)%	75(67-82)%	0.021
2 yr	87(79-91)%	62(53-70)%	<0.001
3 yr	83(75-89)%	58(48-67)%	<0.001

Supplementary Figure 2. Univariable Cox regression for relapse (A) and overall survival (B). Baseline groups for the reported hazard ratio are female for sex, MAC for conditioning intensity, negative for flow cytometry MRD, peripheral blood for graft type, matched unrelated for donor group, 0 for hematopoietic cell transplant specific comorbidity index (HCT-CI), <90 for Karnofsky score, no for ATG, white for race, favorable for ELN, *de novo for* AML Group, negative for baseline *FLT3*-ITD, and negative for baseline *NPM1*.

A

Univariate Cox Regression for Relapse

	hazard ratio	p−value
sex:male	0.954 (0.568−1.6)	0.86
conditioning:RIC/NMA	1.049 (0.626−1.756)	0.86
conditioning3:NMA	1.765 (0.806−3.866)	0.16
conditioning3:RICnoMel	0.999 (0.488−2.047)	1
conditioning3:RICwMel	0.839 (0.417−1.687)	0.62
flowMRD:flowMRDpositive	1.145 (0.491−2.672)	0.75
GraftType:CordBlood	2.33 (1.199−4.529)	0.013
GraftType:BoneMarrow	1.548 (0.789−3.036)	0.2
DonorGroup:MatchedUnrelated	0.911 (0.426−1.947)	0.81
DonorGroup:HaploidentRelated	0.996 (0.301−3.296)	0.99
DonorGroup:CordBlood	1.93 (0.735−5.068)	0.18
DonorGroup:Mismatched	1.48 (0.522−4.196)	0.46
DonorGroup:MultiDonor	2.602 (0.784−8.636)	0.12
HCTCI:1,2	0.744 (0.359−1.543)	0.43
HCTCI:3+	0.94 (0.495−1.785)	0.85
KarnofskyScore:>=90	1.251 (0.732−2.138)	0.41
ATG:yes	1.508 (0.849−2.68)	0.16
race:Other	1.522 (0.791−2.928)	0.21
ELN:adverse	1.719 (0.703−4.205)	0.23
ELN:intermediate	1.903 (0.787−4.599)	0.15
amlGroup:TherapyLinked	0.454 (0.062−3.314)	0.44
amlGroup:TransformedMDSMPS	2.009 (0.874−4.619)	0.1
itdbase:1	1.269 (0.723−2.227)	0.41
npm1base:1	0.915 (0.513−1.633)	0.76

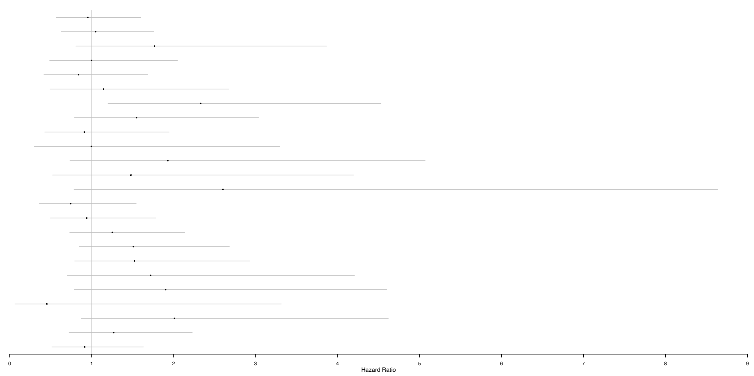

B

Univariate Cox Regression for OS

	hazard ratio	p−value
sexmale	0.963 (0.608−1.525)	0.873
conditioningRIC/NMA	1.395 (0.878−2.217)	0.159
conditioning3NMA	1.863 (0.915−3.793)	0.086
conditioning3RICnoMel	1.531 (0.84−2.792)	0.164
conditioning3RICwMel	1.102 (0.596−2.04)	0.756
flowMRDflowMRDpositive	1.532 (0.758−3.096)	0.235
GraftTypeCordBlood	1.691 (0.876−3.264)	0.117
GraftTypeBoneMarrow	0.988 (0.514−1.899)	0.971
DonorGroupMatchedUnrelated	0.713 (0.369−1.379)	0.315
DonorGroupHaploidentRelated	1.733 (0.727−4.133)	0.215
DonorGroupCordBlood	1.4 (0.569−3.444)	0.464
DonorGroupMismatched	1.195 (0.468−3.051)	0.71
DonorGroupMultiDonor	1.729 (0.486−6.149)	0.398
HCTCI1,2	1.478 (0.642−3.399)	0.358
HCTCI3+	2.778 (1.312−5.882)	0.008
KarnofskyScore>=90	0.703 (0.443−1.116)	0.135
ATGyes	0.929 (0.526−1.641)	0.801
raceOther	1.323 (0.71−2.465)	0.377
ELNadverse	1.174 (0.589−2.34)	0.649
ELNintermediate	0.99 (0.496−1.974)	0.976
amlGroupTherapyLinked	0.713 (0.174−2.919)	0.638
amlGroupTransformedMDSMPS	2.13 (1.05−4.32)	0.036
itdbase	0.777 (0.452−1.339)	0.364
npm1base	0.684 (0.397−1.177)	0.17

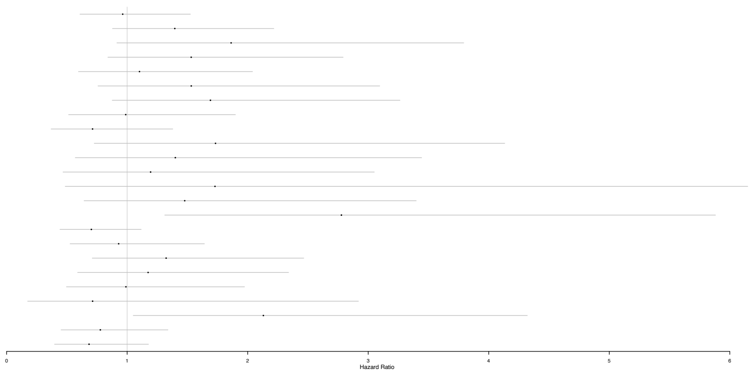

Supplementary Figure 3. Detection of residual variants in pretransplant blood of *IDH2* mutated AML patients during complete remission. (A) NGS MRD assay limit of detection for *IDH2* variants was determined to be 0.1% variant allele fraction (VAF) by serial dilution of three *IDH2* mutations (R140Q, R140W and R172K). The anticipated VAFs of *IDH2* variants were plotted versus the observed ones with a value of equivalence line displayed as a dotted line and the correlations for each types respectively. **(B)** The total number and **(C)** VAF of variants per gene as detected by targeted next-generation sequencing (NGS) during remission prior to transplant in the peripheral blood of *IDH2* mutated AML patients. **(D)** A total of 127 of the 130 detected variants in *IDH2* had a validated assay available for orthogonal validation by digital droplet PCR (ddPCR). The VAFs of *IDH2* variants detected by NGS (x-axis) were plotted versus ddPCR (y-axis), with a value of equivalence line displayed as a dotted line. A significant correlation was observed in the VAF as detected by NGS compared to ddPCR with an orthogonal validation rate of 100%. The Pearson correlation coefficient is shown on the inset of the graph.

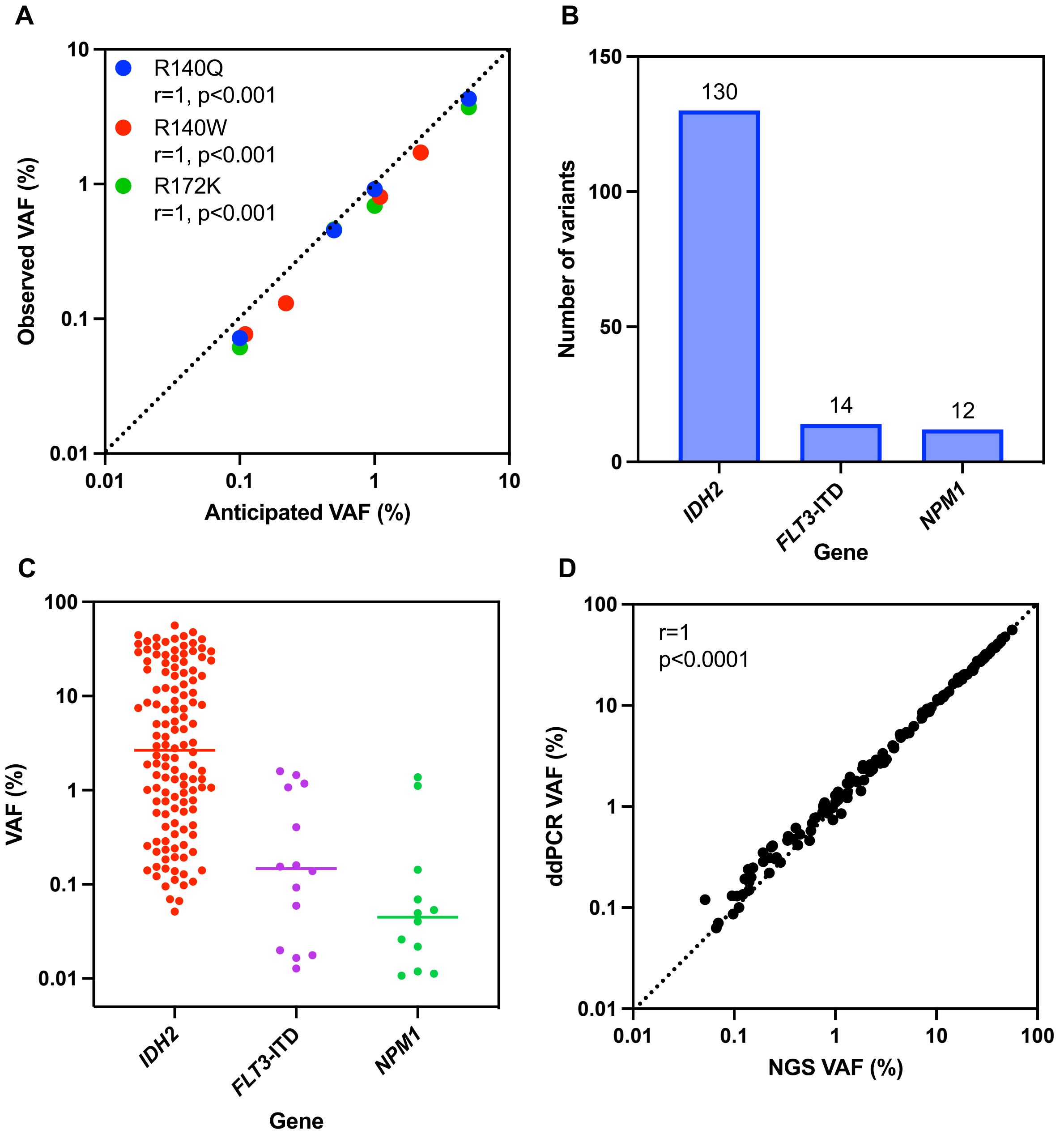

Supplementary Figure 4. NGS MRD status for *IDH2*-mutated AML patients and the association with clinical outcomes after allogeneic hematopoietic cell transplant stratified by age, variant allele fraction (VAF), and mutation type. Cumulative incidence of non-relapse mortality (NRM, top left) and relapse (top right), relapse-free survival (RFS, bottom left) and overall survival (OS, bottom right) shown at 36 months based on *IDH2* NGS-MRD status for **(A)** patients <60 years old, **(B)** patients ≥60 years old, **(C)** NGS MRD *IDH2*+ patients by VAF groups (0%<VAF<2.5%, VAF≥2.5%), and **(D)** NGS MRD *IDH2*+ mutations types (negative, positive with Arg 140, positive Arg 172). Point estimates at different time points are shown in the table (far right). Overall P values: Gray's test for non-relapse mortality (NRM) and relapse; log-rank test for relapse-free survival (RFS) and overall survival (OS). P values for pointwise estimations at different time points: z-test. Confidence interval, CI; Probability, prob; Month, mo; Year, yr; pos, positive; neg, negative.

A

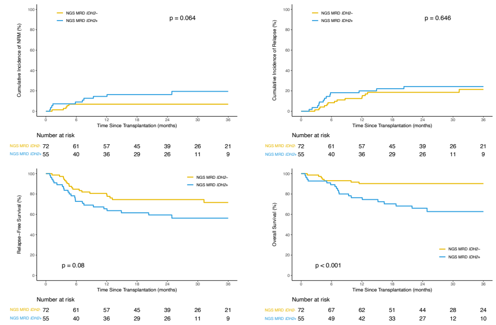

Patients Age < 60 Years Old
NGS MRD *IDH2* Status

Outcomes	Prob (95% CI)	Prob (95% CI)	p-value
NRM	NGS MRD *IDH2*- n=72	NGS MRD *IDH2*+ n=55	0.064
3 mo	1(0-4)%	7(0-14)%	0.121
6 mo	7(1-13)%	9(1-17)%	0.664
1 yr	7(1-13)%	15(5-24)%	0.181
2 yr	7(1-13)%	16(6-26)%	0.109
3 yr	7(1-13)%	19(8-31)%	0.055
Relapse	NGS MRD *IDH2*- n=72	NGS MRD *IDH2*+ n=55	0.646
3 mo	1(0-4)%	4(0-9)%	0.439
6 mo	8(2-15)%	18(8-28)%	0.112
1 yr	13(5-20)%	20(9-31)%	0.265
2 yr	19(9-28)%	24(13-36)%	0.453
3 yr	21(11-32)%	24(13-36)%	0.724
RFS	NGS MRD *IDH2*- n=72	NGS MRD *IDH2*+ n=55	0.08
3 mo	97(89-99)%	89(77-95)%	0.079
6 mo	85(74-91)%	73(59-83)%	0.103
1 yr	81(69-88)%	65(51-76)%	0.057
2 yr	75(63-83)%	59(45-71)%	0.075
3 yr	72(59-81)%	56(41-69)%	0.091
OS	NGS MRD *IDH2*- n=72	NGS MRD *IDH2*+ n=55	<0.001
3 mo	99(91-100)%	93(82-97)%	0.118
6 mo	93(84-97)%	89(77-95)%	0.442
1 yr	90(81-95)%	76(63-86)%	0.039
2 yr	90(81-95)%	66(51-77)%	0.001
3 yr	90(81-95)%	63(47-75)%	<0.001

B

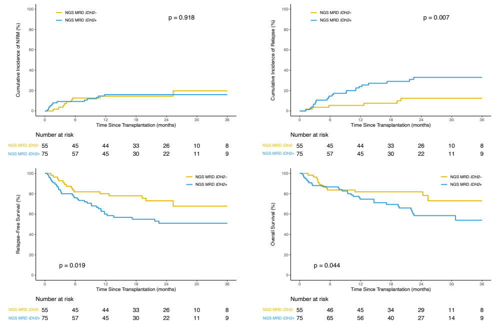

Patients Age ≥ 60 Years Old
NGS MRD *IDH2* Status

Outcomes	Prob (95% CI)	Prob (95% CI)	p-value
NRM	NGS MRD *IDH2*- n=55	NGS MRD *IDH2*+ n=75	0.918
3 mo	4(0-9)%	9(3-16)%	0.179
6 mo	13(4-22)%	9(3-16)%	0.549
1 yr	15(5-24)%	16(8-24)%	0.821
2 yr	15(5-24)%	16(8-24)%	0.821
3 yr	20(6-33)%	16(8-24)%	0.643
Relapse	NGS MRD *IDH2*- n=55	NGS MRD *IDH2*+ n=75	0.007
3 mo	4(0-9)%	7(1-12)%	0.433
6 mo	5(0-12)%	15(7-23)%	0.074
1 yr	5(0-12)%	24(14-34)%	0.002
2 yr	12(3-22)%	33(22-44)%	0.007
3 yr	12(3-22)%	33(22-44)%	0.007
RFS	NGS MRD *IDH2*- n=55	NGS MRD *IDH2*+ n=75	0.019
3 mo	93(82-97)%	84(74-91)%	0.112
6 mo	82(69-90)%	76(65-84)%	0.417
1 yr	80(67-88)%	60(48-70)%	0.011
2 yr	73(58-83)%	51(39-62)%	0.012
3 yr	68(50-80)%	51(39-62)%	0.087
OS	NGS MRD *IDH2*- n=55	NGS MRD *IDH2*+ n=75	0.044
3 mo	96(86-99)%	88(78-94)%	0.064
6 mo	84(71-91)%	87(77-93)%	0.633
1 yr	82(69-90)%	75(63-83)%	0.323
2 yr	82(69-90)%	58(45-69)%	0.004
3 yr	73(55-85)%	54(39-67)%	0.068

C

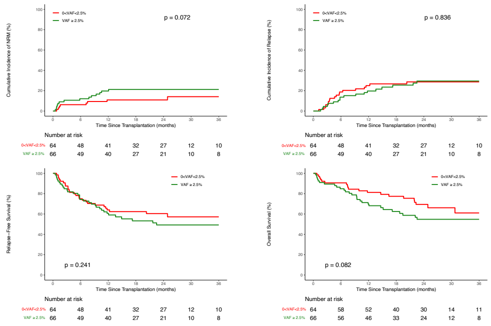

NGS MRD *IDH2*+, Different VAF Groups

Outcomes	Prob (95% CI)	Prob (95% CI)	p-value
NRM	NGS MRD *IDH2*+ 0<VAF<2.5%, n=64	NGS MRD *IDH2*+ VAF ≥ 2.5%, n=66	0.072
3 mo	6(0-12)%	11(3-18)%	0.373
6 mo	6(0-12)%	12(4-20)%	0.247
1 yr	11(3-19)%	20(10-29)%	0.166
2 yr	11(3-19)%	21(11-31)%	0.109
3 yr	14(4-24)%	21(11-31)%	0.316
Relapse	NGS MRD *IDH2*+ 0<VAF<2.5%, n=64	NGS MRD *IDH2*+ VAF ≥ 2.5%, n=66	0.836
3 mo	6(0-12)%	5(0-10)%	0.67
6 mo	19(9-28)%	14(5-22)%	0.432
1 yr	25(14-36)%	20(10-29)%	0.472
2 yr	29(17-40)%	30(18-41)%	0.921
3 yr	29(17-40)%	30(18-41)%	0.921
RFS	NGS MRD *IDH2*+ 0<VAF<2.5%, n=64	NGS MRD *IDH2*+ VAF ≥ 2.5%, n=66	0.241
3 mo	88(77-94)%	85(74-92)%	0.661
6 mo	75(62-84)%	74(62-83)%	0.921
1 yr	64(51-74)%	61(48-71)%	0.684
2 yr	60(47-71)%	49(36-61)%	0.213
3 yr	57(43-69)%	49(36-61)%	0.389
OS	NGS MRD *IDH2*+ 0<VAF<2.5%, n=64	NGS MRD *IDH2*+ VAF ≥ 2.5%, n=66	0.082
3 mo	91(80-96)%	89(79-95)%	0.815
6 mo	91(80-96)%	85(74-92)%	0.313
1 yr	81(69-89)%	70(57-79)%	0.122
2 yr	69(56-80)%	55(41-66)%	0.099
3 yr	61(44-74)%	55(41-66)%	0.538

D

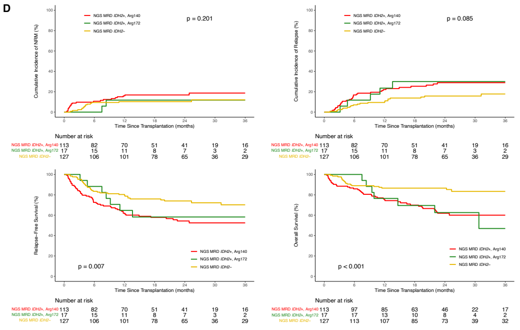

NGS MRD *IDH2* Status by Mutation Type

Outcomes	Prob (95% CI)	Prob (95% CI)	Prob (95% CI)	p-value
NRM	NGS MRD *IDH2*+ Arg140, n=113	NGS MRD *IDH2*+ Arg172, n=17	NGS MRD *IDH2*- n=127	0.201
3 mo	10(4-15)%	0(0-0)%	2(0-5)%	<0.001
6 mo	11(5-16)%	0(0-0)%	9(4-15)%	<0.001
1 yr	16(9-23)%	12(0-28)%	10(5-16)%	0.43
2 yr	17(10-24)%	12(0-28)%	10(5-16)%	0.333
3 yr	19(11-26)%	12(0-28)%	12(6-18)%	0.398
Relapse	NGS MRD *IDH2*+ Arg140, n=113	NGS MRD *IDH2*+ Arg172, n=17	NGS MRD *IDH2*- n=127	0.085
3 mo	6(2-11)%	0(0-0)%	2(0-5)%	0.005
6 mo	17(10-24)%	12(0-28)%	7(3-12)%	0.068
1 yr	22(14-30)%	24(3-44)%	9(4-15)%	0.017
2 yr	29(20-38)%	30(7-53)%	16(9-22)%	0.048
3 yr	29(20-38)%	30(7-53)%	18(10-25)%	0.141
RFS	NGS MRD *IDH2*+ Arg140, n=113	NGS MRD *IDH2*+ Arg172, n=17	NGS MRD *IDH2*- n=127	0.007
3 mo	84(76-90)%	100%	95(90-98)%	<0.001
6 mo	73(63-80)%	88(61-97)%	83(76-89)%	0.069
1 yr	62(52-70)%	65(38-82)%	80(72-86)%	0.005
2 yr	54(44-63)%	58(32-78)%	74(65-81)%	0.006
3 yr	52(42-62)%	58(32-78)%	70(60-78)%	0.032
OS	NGS MRD *IDH2*+ Arg140, n=113	NGS MRD *IDH2*+ Arg172, n=17	NGS MRD *IDH2*- n=127	<0.001
3 mo	88(81-93)%	100%	98(93-99)%	<0.001
6 mo	86(78-91)%	100%	89(82-93)%	<0.001
1 yr	75(66-82)%	76(49-90)%	87(79-91)%	0.069
2 yr	62(52-71)%	63(35-81)%	87(79-91)%	<0.001
3 yr	60(49-69)%	47(15-74)%	83(75-89)%	<0.001

Supplementary Figure 5. NGS MRD status for *IDH2*-mutated AML patients without *NPM1*/*FLT3*-ITD mutations at baseline and the association with clinical outcomes after allogeneic hematopoietic cell transplant stratified by age group. Cumulative incidence of non-relapse mortality (NRM, top left) and relapse (top right), relapse-free survival (RFS, bottom left) and overall survival (OS, bottom right) shown at 36 months for **(A)** all *IDH2*-mutated AML patients without *NPM1* or *FLT3*-ITD mutations at baseline, **(B)** *IDH2*-mutated AML patients without *NPM1* or *FLT3*-ITD mutations at baseline aged below 60, and **(C)** *IDH2*-mutated AML patients without *NPM1* or *FLT3*-ITD mutations at baseline aged 60 and above. Point estimates at different time points are shown in the table (far right). Overall P values: Gray's test for non-relapse mortality (NRM) and relapse; log-rank test for relapse-free survival (RFS) and overall survival (OS). P values for pointwise estimations at different time points: z-test. Confidence interval, CI; Probability, prob; Month, mo; Year, yr; pos, positive; neg, negative.

A

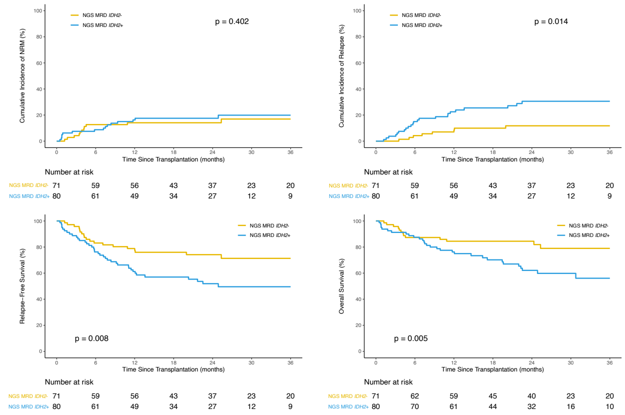

No *NPM1* and/or *FLT3*-ITD at Baseline
NGS MRD *IDH2* Status

Outcomes	Prob (95% CI)	Prob (95% CI)	p-value
NRM	NGS MRD *IDH2*- n=71	NGS MRD *IDH2*+ n=80	0.402
3 mo	4(0-9)%	8(2-13)%	0.391
6 mo	13(5-20)%	9(3-15)%	0.441
1 yr	14(6-22)%	16(8-24)%	0.713
2 yr	14(6-22)%	18(9-26)%	0.565
3 yr	17(7-27)%	20(10-29)%	0.668
Relapse	NGS MRD *IDH2*- n=71	NGS MRD *IDH2*+ n=80	0.014
3 mo	0(0-0)%	4(0-8)%	0.08
6 mo	4(0-9)%	15(7-23)%	0.022
1 yr	7(1-13)%	23(13-32)%	0.006
2 yr	12(4-20)%	31(20-41)%	0.005
3 yr	12(4-20)%	31(20-41)%	0.005
RFS	NGS MRD *IDH2*- n=71	NGS MRD *IDH2*+ n=80	0.008
3 mo	96(87-99)%	89(79-94)%	0.099
6 mo	83(72-90)%	76(65-84)%	0.293
1 yr	79(67-87)%	61(50-71)%	0.016
2 yr	74(62-83)%	52(40-63)%	0.005
3 yr	71(58-81)%	50(37-61)%	0.009
OS	NGS MRD *IDH2*- n=71	NGS MRD *IDH2*+ n=80	0.005
3 mo	96(87-99)%	91(83-96)%	0.253
6 mo	87(77-93)%	88(78-93)%	0.974
1 yr	84(74-91)%	76(65-84)%	0.201
2 yr	84(74-91)%	62(50-72)%	0.002
3 yr	79(66-88)%	56(42-68)%	0.008

B

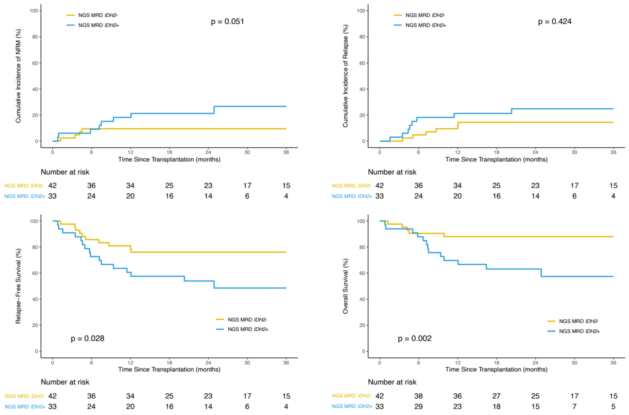

No *NPM1* and/or *FLT3*-ITD at Baseline
Patient Age < 60 Years Old
NGS MRD *IDH2* Status

Outcomes	Prob (95% CI)	Prob (95% CI)	p-value
NRM	NGS MRD *IDH2*- n=42	NGS MRD *IDH2*+ n=33	0.051
3 mo	2(0-7)%	6(0-14)%	0.448
6 mo	10(1-19)%	9(0-19)%	0.95
1 yr	10(1-19)%	18(5-32)%	0.294
2 yr	10(1-19)%	21(7-35)%	0.174
3 yr	10(1-19)%	27(10-44)%	0.082
Relapse	NGS MRD *IDH2*- n=42	NGS MRD *IDH2*+ n=33	0.424
3 mo	0(0-0)%	3(0-9)%	0.318
6 mo	5(0-11)%	18(5-32)%	0.078
1 yr	10(1-19)%	21(7-35)%	0.173
2 yr	14(4-25)%	25(9-40)%	0.279
3 yr	14(4-25)%	25(9-40)%	0.279
RFS	NGS MRD *IDH2*- n=42	NGS MRD *IDH2*+ n=33	0.028
3 mo	98(84-100)%	91(74-97)%	0.225
6 mo	86(71-93)%	73(54-85)%	0.169
1 yr	81(66-90)%	61(42-75)%	0.051
2 yr	76(60-86)%	54(36-69)%	0.045
3 yr	76(60-86)%	49(29-65)%	0.017
OS	NGS MRD *IDH2*- n=42	NGS MRD *IDH2*+ n=33	0.002
3 mo	98(84-100)%	94(78-98)%	0.441
6 mo	90(77-96)%	88(71-95)%	0.721
1 yr	88(74-95)%	70(51-82)%	0.052
2 yr	88(74-95)%	63(44-77)%	0.012
3 yr	88(74-95)%	57(37-73)%	0.004

C

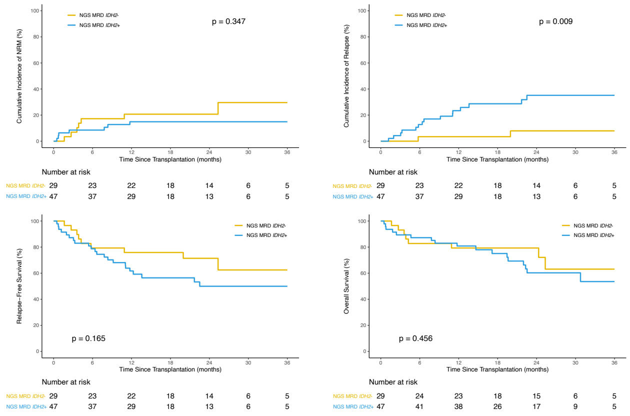

No *NPM1* and/or *FLT3*-ITD at Baseline
Patient Age ≥ 60 Years Old
NGS MRD *IDH2* Status

Outcomes	Prob (95% CI)	Prob (95% CI)	p-value
NRM	NGS MRD *IDH2*- n=29	NGS MRD *IDH2*+ n=47	0.347
3 mo	7(0-16)%	9(0-17)%	0.798
6 mo	17(3-31)%	9(0-17)%	0.29
1 yr	21(6-36)%	15(5-25)%	0.533
2 yr	21(6-36)%	15(5-25)%	0.533
3 yr	30(8-52)%	15(5-25)%	0.236
Relapse	NGS MRD *IDH2*- n=29	NGS MRD *IDH2*+ n=47	0.009
3 mo	0(0-0)%	4(0-10)%	0.153
6 mo	3(0-10)%	13(3-22)%	0.122
1 yr	3(0-10)%	23(11-36)%	0.005
2 yr	8(0-19)%	35(20-50)%	0.004
3 yr	8(0-19)%	35(20-50)%	0.004
RFS	NGS MRD *IDH2*- n=29	NGS MRD *IDH2*+ n=47	0.165
3 mo	93(75-98)%	87(74-94)%	0.386
6 mo	79(60-90)%	79(64-88)%	0.951
1 yr	76(56-88)%	62(46-74)%	0.184
2 yr	71(51-85)%	50(34-64)%	0.066
3 yr	62(37-80)%	50(34-64)%	0.362
OS	NGS MRD *IDH2*- n=29	NGS MRD *IDH2*+ n=47	0.456
3 mo	93(75-98)%	89(76-95)%	0.565
6 mo	83(63-92)%	87(74-94)%	0.6
1 yr	79(60-90)%	81(66-90)%	0.871
2 yr	79(60-90)%	60(43-74)%	0.082
3 yr	63(36-81)%	54(34-70)%	0.533

Supplementary Figure 6. NGS MRD status for *IDH2*-mutated AML patients with *NPM1*/*FLT3*-ITD mutations at baseline and the association with clinical outcomes after allogeneic hematopoietic cell transplant. Cumulative incidence of non-relapse mortality (NRM, top left) and relapse (top right), relapse-free survival (RFS, bottom left) and overall survival (OS, bottom right) shown at 36 months for all *IDH2*-mutated AML patients with *NPM1* and/or *FLT3*-ITD mutations at baseline, with NGS MRD information including **(A)** only NGS MRD *IDH2* status, **(B)** *NPM1* and/or *FLT3*-ITD positive (regardless of NGS MRD *IDH2* status, NGS MRD *NPM1*/*FLT3*-ITD+), both *NPM1* and *FLT3*-ITD negative with *IDH2* positive (NGS MRD IDH2+ only). Point estimates at different time points are shown in the table (far right). Overall P values: Gray's test for non-relapse mortality (NRM) and relapse; log-rank test for relapse-free survival (RFS) and overall survival (OS). P values for pointwise estimations at different time points: z-test. Confidence interval, CI; Probability, prob; Month, mo; Year, yr; pos, positive; neg, negative.

A

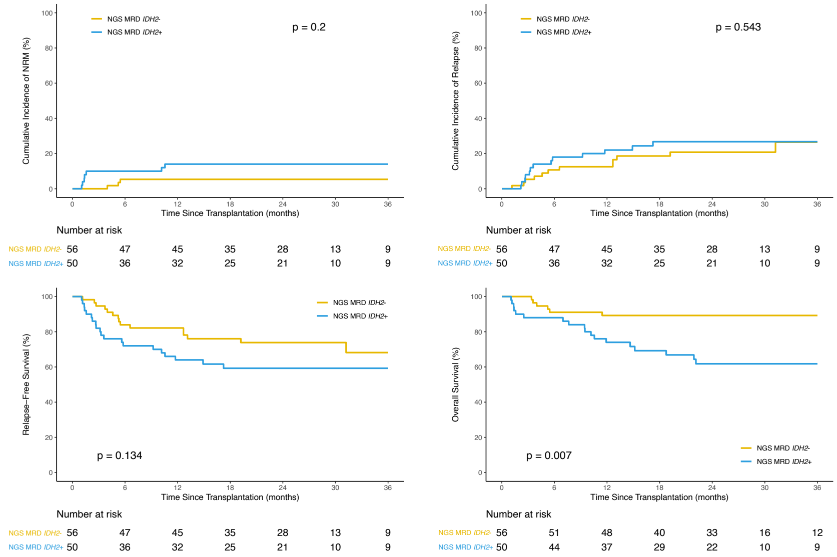

Patients with *NPM1* and/or *FLT3*-ITD at Baseline NGS MRD Status

Outcomes	Prob (95% CI)	Prob (95% CI)	p-value
NRM	NGS MRD *IDH2*- n=56	NGS MRD *IDH2+* n=50	0.2
3 mo	0(0-0)%	10(2-18)%	0.02
6 mo	5(0-11)%	10(2-18)%	0.377
1 yr	5(0-11)%	14(4-24)%	0.138
2 yr	5(0-11)%	14(4-24)%	0.138
3 yr	5(0-11)%	14(4-24)%	0.138
Relapse	NGS MRD *IDH2*- n=56	NGS MRD *IDH2+* n=50	0.543
3 mo	5(0-11)%	8(0-16)%	0.592
6 mo	11(3-19)%	18(7-29)%	0.291
1 yr	12(4-21)%	22(10-34)%	0.201
2 yr	21(10-32)%	27(14-39)%	0.49
3 yr	26(11-42)%	27(14-39)%	0.979
RFS	NGS MRD *IDH2*- n=56	NGS MRD *IDH2+* n=50	0.134
3 mo	95(84-98)%	82(68-90)%	0.042
6 mo	84(71-91)%	72(57-82)%	0.137
1 yr	82(69-90)%	64(49-76)%	0.033
2 yr	74(60-84)%	59(44-72)%	0.117
3 yr	68(50-81)%	59(44-72)%	0.398
OS	NGS MRD *IDH2*- n=56	NGS MRD *IDH2+* n=50	0.007
3 mo	100%	88(75-94)%	<0.001
6 mo	91(80-96)%	88(75-94)%	0.607
1 yr	89(78-95)%	74(59-84)%	0.041
2 yr	89(78-95)%	62(46-74)%	<0.001
3 yr	89(78-95)%	62(46-74)%	<0.001

B

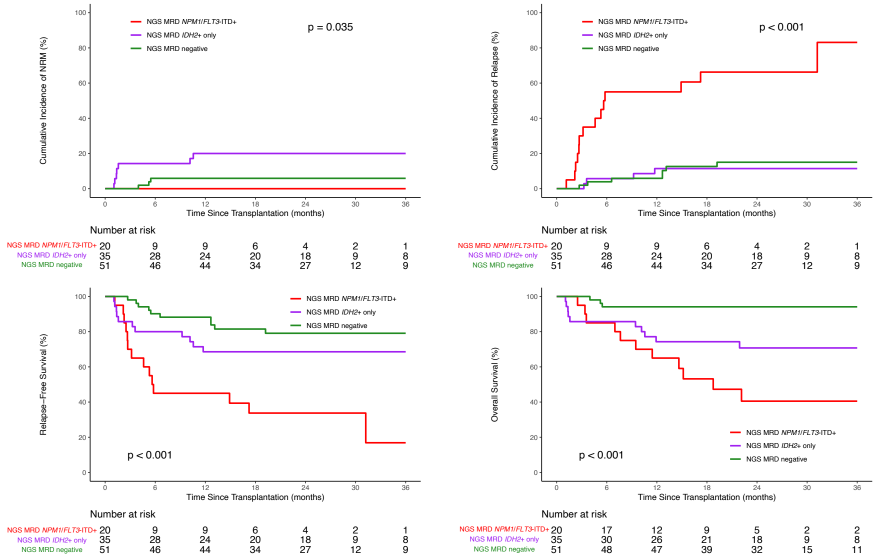

Patients with *NPM1* and/or *FLT3*-ITD at Baseline NGS MRD Status

Outcomes	Prob (95% CI)	Prob (95% CI)	Prob (95% CI)	p-value
NRM	NGS MRD *NPM1*/*FLT3*-ITD positive=20	NGS MRD *IDH2* positive only=35	NGS MRD negative=51	0.035
3 mo	0(0-0)%	14(3-26)%	0(0-0)%	NA
6 mo	0(0-0)%	14(3-26)%	6(0-12)%	0.012
1 yr	0(0-0)%	20(7-33)%	6(0-12)%	0.003
2 yr	0(0-0)%	20(7-33)%	6(0-12)%	0.003
3 yr	0(0-0)%	20(7-33)%	6(0-12)%	0.003
Relapse	NGS MRD *NPM1*/*FLT3*-ITD positive=20	NGS MRD *IDH2* positive only=35	NGS MRD negative=51	<0.001
3 mo	30(9-51)%	0(0-0)%	2(0-6)%	0.011
6 mo	55(32-78)%	6(0-14)%	4(0-9)%	<0.001
1 yr	55(32-78)%	11(1-22)%	6(0-12)%	<0.001
2 yr	66(44-89)%	11(1-22)%	15(5-25)%	<0.001
3 yr	83(48-100)%	11(1-22)%	15(5-25)%	<0.001
RFS	NGS MRD *NPM1*/*FLT3*-ITD positive=20	NGS MRD *IDH2* positive only=35	NGS MRD negative=51	<0.001
3 mo	70(45-85)%	86(69-94)%	98(87-100)%	0.005
6 mo	45(23-65)%	80(63-90)%	90(78-96)%	<0.001
1 yr	45(23-65)%	69(50-81)%	88(76-95)%	<0.001
2 yr	34(14-54)%	69(50-81)%	79(64-88)%	0.001
3 yr	17(2-47)%	69(50-81)%	79(64-88)%	<0.001
OS	NGS MRD *NPM1*/*FLT3*-ITD positive=20	NGS MRD *IDH2* positive only=35	NGS MRD negative=51	<0.001
3 mo	95(69-99)%	86(69-94)%	100%	<0.001
6 mo	85(60-95)%	86(69-94)%	94(83-98)%	0.325
1 yr	65(40-82)%	74(56-86)%	94(83-98)%	0.003
2 yr	41(18-62)%	71(52-83)%	94(83-98)%	<0.001
3 yr	41(18-62)%	71(52-83)%	94(83-98)%	<0.001

Supplementary Figure 7. NGS MRD status for *IDH2*-mutated AML patients and the association with clinical outcomes after allogeneic hematopoietic cell transplant stratified by conditioning intensity and age group. Cumulative incidence of non-relapse mortality (NRM, top left) and relapse (top right), relapse-free survival (RFS, bottom left) and overall survival (OS, bottom right) shown at 36 months based on NGS MRD *IDH2* status and **(A)** conditioning intensity, **(B)** conditioning intensity for patients 60 and above. MAC/mel: myeloablative conditioning or reduced intensity conditioning with melphalan; RIC/NMA: reduced intensity conditioning without melphalan or non-myeloablative conditioning. Point estimates at different time points are shown in the table (far right). Overall P values: Gray's test for non-relapse mortality (NRM) and relapse; log-rank test for relapse-free survival (RFS) and overall survival (OS). P values for pointwise estimations at different time points: z-test. Confidence interval, CI; Probability, prob; Month, mo; Year, yr; pos, positive; neg, negative.

A

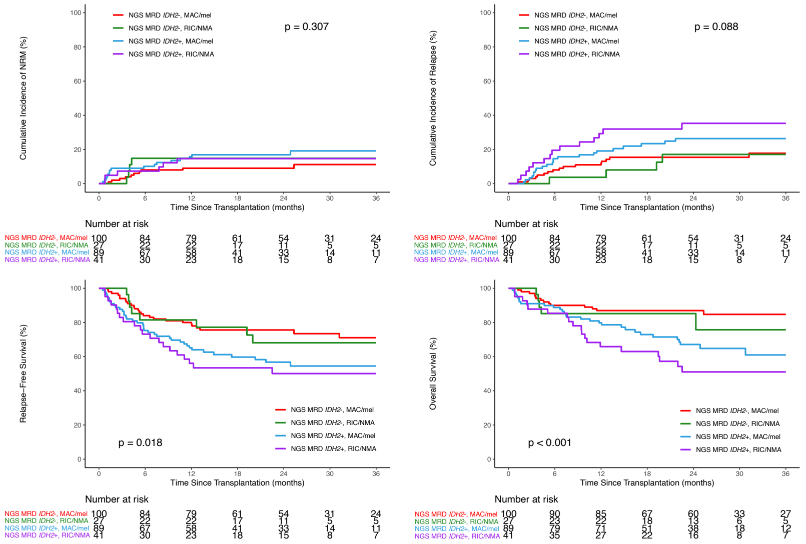

NGS MRD *IDH2* Status and Conditioning Intensity

Outcomes	Prob (95% CI)	Prob (95% CI)	Prob (95% CI)	Prob (95% CI)	p-value
NRM	NGS MRD *IDH2*- MAC/mel=100	NGS MRD *IDH2*- RIC/NMA=27	NGS MRD *IDH2*+ MAC/mel=89	NGS MRD *IDH2*+ RIC/NMA=41	0.307
3 mo	3(0-6)%	0(0-0)%	9(3-15)%	7(0-15)%	0.002
6 mo	8(3-13)%	15(1-28)%	10(4-16)%	7(0-15)%	0.773
1 yr	9(3-15)%	15(1-28)%	16(8-23)%	15(4-26)%	0.496
2 yr	9(3-15)%	15(1-28)%	17(9-25)%	15(4-26)%	0.403
3 yr	11(4-18)%	15(1-28)%	19(10-28)%	15(4-26)%	0.583
Relapse	NGS MRD *IDH2*- MAC/mel=100	NGS MRD *IDH2*- RIC/NMA=27	NGS MRD *IDH2*+ MAC/mel=89	NGS MRD *IDH2*+ RIC/NMA=41	0.088
3 mo	3(0-6)%	0(0-0)%	3(0-7)%	10(1-19)%	0.015
6 mo	8(3-13)%	4(0-11)%	15(7-22)%	20(7-32)%	0.068
1 yr	11(5-17)%	4(0-11)%	19(11-27)%	29(15-43)%	0.003
2 yr	15(8-23)%	17(1-33)%	26(17-36)%	35(20-51)%	0.071
3 yr	18(9-26)%	17(1-33)%	26(17-36)%	35(20-51)%	0.177
RFS	NGS MRD *IDH2*- MAC/mel=100	NGS MRD *IDH2*- RIC/NMA=27	NGS MRD *IDH2*+ MAC/mel=89	NGS MRD *IDH2*+ RIC/NMA=41	0.018
3 mo	94(87-97)%	100%	88(79-93)%	83(67-91)%	<0.001
6 mo	84(75-90)%	81(61-92)%	75(65-83)%	73(57-84)%	0.354
1 yr	80(71-87)%	81(61-92)%	65(54-74)%	56(40-70)%	0.009
2 yr	76(66-83)%	68(46-83)%	57(45-67)%	50(34-64)%	0.008
3 yr	71(60-80)%	68(46-83)%	54(43-65)%	50(34-64)%	0.058
OS	NGS MRD *IDH2*- MAC/mel=100	NGS MRD *IDH2*- RIC/NMA=27	NGS MRD *IDH2*+ MAC/mel=89	NGS MRD *IDH2*+ RIC/NMA=41	<0.001
3 mo	97(91-99)%	100%	91(83-95)%	88(73-95)%	<0.001
6 mo	90(82-94)%	85(65-94)%	89(80-94)%	85(70-93)%	0.846
1 yr	87(79-92)%	85(65-94)%	80(70-87)%	66(49-78)%	0.061
2 yr	87(79-92)%	85(65-94)%	67(56-76)%	51(34-66)%	<0.001
3 yr	85(75-91)%	76(47-90)%	61(47-72)%	51(34-66)%	<0.001

B

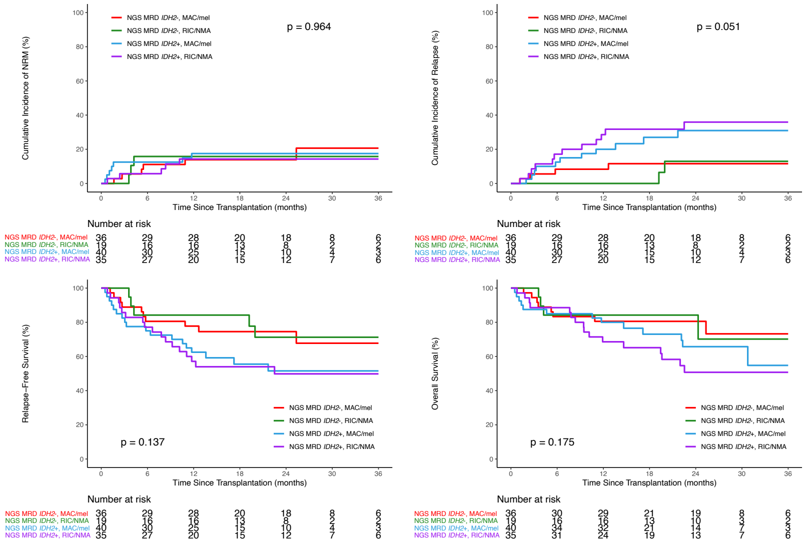

Patients Age ≥ 60 Years Old
NGS MRD *IDH2* Status and Conditioning Intensity

Outcomes	Prob (95% CI)	Prob (95% CI)	Prob (95% CI)	Prob (95% CI)	p-value
NRM	NGS MRD *IDH2*- MAC/mel=36	NGS MRD *IDH2*- RIC/NMA=19	NGS MRD *IDH2*+ MAC/mel=40	NGS MRD *IDH2*+ RIC/NMA=35	0.964
3 mo	6(0-13)%	0(0-0)%	12(2-23)%	6(0-14)%	0.022
6 mo	11(1-22)%	16(0-33)%	12(2-23)%	6(0-14)%	0.604
1 yr	14(2-25)%	16(0-33)%	18(6-29)%	14(2-26)%	0.974
2 yr	14(2-25)%	16(0-33)%	18(6-29)%	14(2-26)%	0.974
3 yr	21(4-38)%	16(0-33)%	18(6-29)%	14(2-26)%	0.941
Relapse	NGS MRD *IDH2*- MAC/mel=36	NGS MRD *IDH2*- RIC/NMA=19	NGS MRD *IDH2*+ MAC/mel=40	NGS MRD *IDH2*+ RIC/NMA=35	0.051
3 mo	6(0-13)%	0(0-0)%	5(0-12)%	9(0-18)%	0.063
6 mo	8(0-18)%	0(0-0)%	12(2-23)%	17(4-30)%	0.001
1 yr	8(0-18)%	0(0-0)%	20(7-33)%	29(13-44)%	<0.001
2 yr	12(1-22)%	13(0-30)%	31(15-47)%	36(19-53)%	0.045
3 yr	12(1-22)%	13(0-30)%	31(15-47)%	36(19-53)%	0.045
RFS	NGS MRD *IDH2*- MAC/mel=36	NGS MRD *IDH2*- RIC/NMA=19	NGS MRD *IDH2*+ MAC/mel=40	NGS MRD *IDH2*+ RIC/NMA=35	0.137
3 mo	89(73-96)%	100%	82(67-91)%	86(69-94)%	<0.001
6 mo	81(63-90)%	84(59-95)%	75(59-86)%	77(59-88)%	0.837
1 yr	78(60-88)%	84(59-95)%	62(46-75)%	57(39-72)%	0.06
2 yr	75(57-86)%	71(44-87)%	52(34-67)%	50(32-65)%	0.071
3 yr	68(46-82)%	71(44-87)%	52(34-67)%	50(32-65)%	0.265
OS	NGS MRD *IDH2*- MAC/mel=36	NGS MRD *IDH2*- RIC/NMA=19	NGS MRD *IDH2*+ MAC/mel=40	NGS MRD *IDH2*+ RIC/NMA=35	0.175
3 mo	94(80-99)%	100%	88(73-95)%	89(72-96)%	<0.001
6 mo	83(67-92)%	84(59-95)%	85(70-93)%	89(72-96)%	0.924
1 yr	81(63-90)%	84(59-95)%	80(64-89)%	69(50-81)%	0.528
2 yr	81(63-90)%	84(59-95)%	66(47-79)%	51(32-67)%	0.019
3 yr	73(50-87)%	70(33-89)%	55(29-75)%	51(32-67)%	0.295

Supplementary Figure 8. Patient transplant years for *IDH2*-mutated AML patients and the association with clinical outcomes after allogeneic hematopoietic cell transplant stratified by NGS MRD status and baseline mutation. Cumulative incidence of non-relapse mortality (NRM, top left) and relapse (top right), relapse-free survival (RFS, bottom left) and overall survival (OS, bottom right) shown at 36 months based on *IDH2* NGS-MRD status based on year of transplantation (before or after August 2017) for **(A)** patients stratified by *IDH2* NGS-MRD status, and **(B)** patients without baseline *FLT3*-ITD mutation reported stratified by *IDH2* NGS-MRD status. Point estimates at different time points are shown in the table (far right). Overall P values: Gray's test for non-relapse mortality (NRM) and relapse; log-rank test for relapse-free survival (RFS) and overall survival (OS). P values for pointwise estimations at different time points: z-test. Confidence interval, CI; Probability, prob; Month, mo; Year, yr; pos, positive; neg, negative.

A

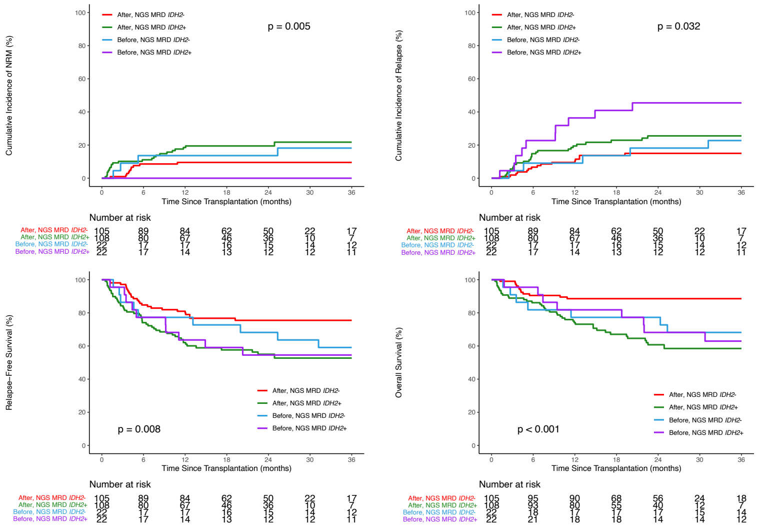

Patients having alloHCT Before or After August 2017
NGS MRD *IDH2* Status

Outcomes	Prob (95% CI)	Prob (95% CI)	Prob (95% CI)	Prob (95% CI)	p-value
NRM	After NGS MRD *IDH2*- n=105	After NGS MRD *IDH2*+ n=108	Before NGS MRD *IDH2*- n=22	Before NGS MRD *IDH2*+ n=22	0.005
3 mo	1(0-3)%	10(4-16)%	9(0-21)%	0(0-0)%	0.002
6 mo	9(3-14)%	11(5-17)%	14(0-28)%	0(0-0)%	<0.001
1 yr	10(4-15)%	19(11-26)%	14(0-28)%	0(0-0)%	<0.001
2 yr	10(4-15)%	19(12-27)%	14(0-28)%	0(0-0)%	<0.001
3 yr	10(4-15)%	22(13-30)%	18(2-35)%	0(0-0)%	<0.001
Relapse	After NGS MRD *IDH2*- n=105	After NGS MRD *IDH2*+ n=108	Before NGS MRD *IDH2*- n=22	Before NGS MRD *IDH2*+ n=22	0.032
3 mo	2(0-5)%	6(1-10)%	5(0-13)%	5(0-13)%	0.527
6 mo	7(2-11)%	15(8-22)%	9(0-21)%	23(5-41)%	0.124
1 yr	10(4-15)%	19(12-27)%	9(0-21)%	36(16-57)%	0.023
2 yr	15(8-22)%	26(17-34)%	18(2-35)%	45(24-67)%	0.031
3 yr	15(8-22)%	26(17-34)%	23(5-41)%	45(24-67)%	0.032
RFS	After NGS MRD *IDH2*- n=105	After NGS MRD *IDH2*+ n=108	Before NGS MRD *IDH2*- n=22	Before NGS MRD *IDH2*+ n=22	0.008
3 mo	97(91-99)%	84(76-90)%	86(63-95)%	95(72-99)%	0.006
6 mo	85(76-90)%	74(65-81)%	77(54-90)%	77(54-90)%	0.261
1 yr	81(72-87)%	62(52-70)%	77(54-90)%	64(40-80)%	0.012
2 yr	75(66-83)%	55(45-64)%	68(45-83)%	55(32-72)%	0.012
3 yr	75(66-83)%	53(42-62)%	59(36-76)%	55(32-72)%	0.005
OS	After NGS MRD *IDH2*- n=105	After NGS MRD *IDH2*+ n=108	Before NGS MRD *IDH2*- n=22	Before NGS MRD *IDH2*+ n=22	<0.001
3 mo	99(93-100)%	89(81-94)%	91(68-98)%	95(72-99)%	0.008
6 mo	90(83-95)%	86(78-91)%	82(59-93)%	95(72-99)%	0.281
1 yr	89(81-93)%	74(65-81)%	77(54-90)%	82(59-93)%	0.044
2 yr	89(81-93)%	61(50-70)%	77(54-90)%	68(45-83)%	<0.001
3 yr	89(81-93)%	58(47-68)%	68(45-83)%	63(39-80)%	<0.001

B

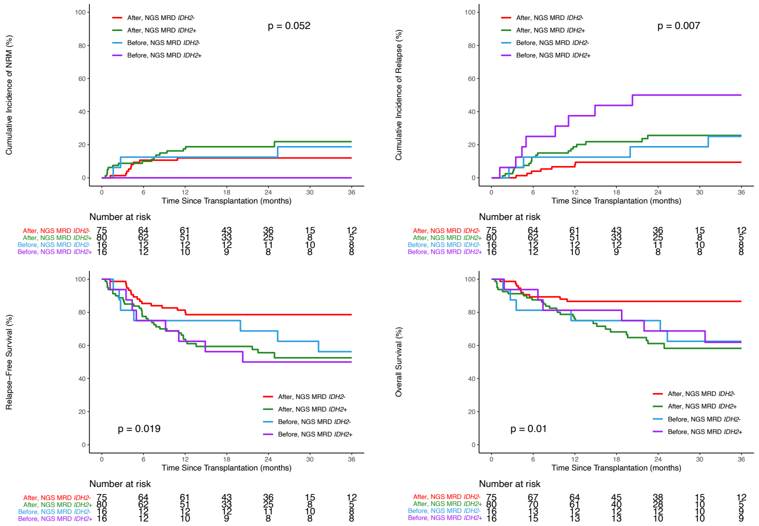

Patients without *FLT3*-ITD Mutations at Baseline
Having alloHCT Before or After August 2017
NGS MRD *IDH2* Status

Outcomes	Prob (95% CI)	Prob (95% CI)	Prob (95% CI)	Prob (95% CI)	p-value
NRM	After NGS MRD *IDH2*- n=75	After NGS MRD *IDH2*+ n=80	Before NGS MRD *IDH2*- n=16	Before NGS MRD *IDH2*+ n=16	0.052
3 mo	1(0-4)%	9(3-15)%	12(0-29)%	0(0-0)%	0.013
6 mo	11(4-18)%	10(3-17)%	12(0-29)%	0(0-0)%	<0.001
1 yr	12(5-19)%	18(9-26)%	12(0-29)%	0(0-0)%	<0.001
2 yr	12(5-19)%	19(10-27)%	12(0-29)%	0(0-0)%	<0.001
3 yr	12(5-19)%	22(12-32)%	19(0-39)%	0(0-0)%	<0.001
Relapse	After NGS MRD *IDH2*- n=75	After NGS MRD *IDH2*+ n=80	Before NGS MRD *IDH2*- n=16	Before NGS MRD *IDH2*+ n=16	0.007
3 mo	0(0-0)%	2(0-6)%	6(0-19)%	6(0-18)%	0.259
6 mo	4(0-8)%	12(5-20)%	12(0-29)%	25(3-47)%	0.076
1 yr	7(1-12)%	19(10-27)%	12(0-29)%	38(13-62)%	0.021
2 yr	9(3-16)%	26(15-36)%	19(0-39)%	50(24-76)%	0.003
3 yr	9(3-16)%	26(15-36)%	25(3-47)%	50(24-76)%	0.002
RFS	After NGS MRD *IDH2*- n=75	After NGS MRD *IDH2*+ n=80	Before NGS MRD *IDH2*- n=16	Before NGS MRD *IDH2*+ n=16	0.019
3 mo	99(91-100)%	89(79-94)%	81(52-94)%	94(63-99)%	0.019
6 mo	85(75-92)%	78(67-85)%	75(46-90)%	75(46-90)%	0.512
1 yr	81(71-88)%	64(52-73)%	75(46-90)%	62(35-81)%	0.067
2 yr	79(67-86)%	56(43-66)%	69(40-86)%	50(25-71)%	0.009
3 yr	79(67-86)%	53(40-64)%	56(30-76)%	50(25-71)%	0.003
OS	After NGS MRD *IDH2*- n=75	After NGS MRD *IDH2*+ n=80	Before NGS MRD *IDH2*- n=16	Before NGS MRD *IDH2*+ n=16	0.01
3 mo	99(91-100)%	91(83-96)%	88(59-97)%	94(63-99)%	0.091
6 mo	89(80-95)%	88(78-93)%	81(52-94)%	94(63-99)%	0.702
1 yr	87(77-93)%	76(65-84)%	75(46-90)%	81(52-94)%	0.353
2 yr	87(77-93)%	61(48-72)%	75(46-90)%	69(40-86)%	0.004
3 yr	87(77-93)%	58(45-69)%	62(35-81)%	62(34-81)%	<0.001

Supplementary Table 1. Variants detected by next-generation sequencing in the blood of *IDH2* mutated AML patients prior to transplant conditioning.

Subject ID	Gene	HGVSp	HGVSc	VAF	Alternate Observations	Deep Alternate Observations	Unique Alternate Observations	Depth	Deep Depth	Variant Consequence	Chromosome	Position	Reference	Alternate	Previously reported*	ddPCR validated	ddPCR VAF
CH 012119	NPM1	NP_002511.1:p.Trp288CysfsTer12	NM_002520.6:c.860_863dup	0.0001	4	1	1	37243	10054	frameshift	5	170837543	C	CTCTG	Yes	Yes	0.00015
CH 012230	IDH2	NP_002159.2:p.Arg140Gln	NM_002168.3:c.419G>A	0.0075	159	89	72	18648	11911	missense	15	90631934	C	T	Yes	Yes	0.00875
CH 012019	IDH2	NP_002159.2:p.Arg172Lys	NM_002168.3:c.515G>A	0.0078	185	101	88	20376	12878	missense	15	90631838	C	T	Yes	Yes	0.01096
CH 012512	IDH2	NP_002159.2:p.Arg140Trp	NM_002168.3:c.418C>T	0.0034	68	33	41	16062	9811	missense	15	90631935	G	A	Yes	Yes	0.00463
CH 012515	IDH2	NP_002159.2:p.Arg140Gln	NM_002168.3:c.419G>A	0.2818	4924	3290	432	17187	11676	missense	15	90631934	C	T	Yes	Yes	0.29130
CH 012541	IDH2	NP_002159.2:p.Arg140Gln	NM_002168.3:c.419G>A	0.0056	131	55	64	18810	9873	missense	15	90631934	C	T	Yes	Yes	0.00460
CH 012100	IDH2	NP_002159.2:p.Arg172Lys	NM_002168.3:c.515G>A	0.0014	31	18	24	19024	12810	missense	15	90631838	C	T	Yes	Yes	0.00149
CH 012323	IDH2	NP_002159.2:p.Arg140Gln	NM_002168.3:c.419G>A	0.0013	51	22	32	26902	17214	missense	15	90631934	C	T	Yes	Yes	0.00192
CH 012329	IDH2	NP_002159.2:p.Arg140Gln	NM_002168.3:c.419G>A	0.3575	5144	3368	469	14452	9421	missense	15	90631934	C	T	Yes	Yes	0.36602
CH 012327	IDH2	NP_002159.2:p.Arg140Gln	NM_002168.3:c.419G>A	0.0019	42	22	26	17620	11313	missense	15	90631934	C	T	Yes	Yes	0.00284
CH 012416	IDH2	NP_002159.2:p.Arg140Gln	NM_002168.3:c.419G>A	0.3664	8997	5320	622	24225	14520	missense	15	90631934	C	T	Yes	Yes	0.37451
CH 012418	NPM1	NP_002511.1:p.Trp288CysfsTer12	NM_002520.6:c.863_864insCCAG	0.0002	7	1	5	32176	4816	frameshift	5	170837546	T	TGCCA	Yes	Not Done	Not Done
CH 012418	FLT3	NP_004110.2:p.Phe594_Asn609dup	NM_004119.2:c.1782_1829dup	0.0001	13		8	101879		FLT3-ITD	13	28608230	T	TTTTCTCTTGGAAACTCCCATTTGAGATCATATTC ATATTCTCTGAAAT	Yes	Not Done	Not Done
CH 012490	IDH2	NP_002159.2:p.Arg140Gln	NM_002168.3:c.419G>A	0.038	973	497	269	23680	13065	missense	15	90631934	C	T	Yes	Yes	0.03784
CH 012568	IDH2	NP_002159.2:p.Arg140Gln	NM_002168.3:c.419G>A	0.0115	163	86	63	14227	7506	missense	15	90631934	C	T	Yes	Yes	0.00849
CH 012044	IDH2	NP_002159.2:p.Arg140Gln	NM_002168.3:c.419G>A	0.0012	28	14	21	17808	11476	missense	15	90631934	C	T	Yes	Yes	0.00135
CH 012554	IDH2	NP_002159.2:p.Arg140Gln	NM_002168.3:c.419G>A	0.4452	7951	4908	472	17801	11024	missense	15	90631934	C	T	Yes	Yes	0.45490
CH 012520	IDH2	NP_002159.2:p.Arg172Lys	NM_002168.3:c.515G>A	0.001	17	10	14	15808	10222	missense	15	90631838	C	T	Yes	Yes	0.00086
CH 012529	IDH2	NP_002159.2:p.Arg140Gln	NM_002168.3:c.419G>A	0.1176	1717	1062	315	14434	9031	missense	15	90631934	C	T	Yes	Yes	0.12136
CH 012551	IDH2	NP_002159.2:p.Arg140Gln	NM_002168.3:c.419G>A	0.0858	1681	1017	313	18724	11853	missense	15	90631934	C	T	Yes	Yes	0.09260
CH 012551	NPM1	NP_002511.1:p.Trp288CysfsTer12	NM_002520.6:c.860_863dup	0.0005	14	0	9	28293	5606	frameshift	5	170837543	C	CTCTG	Yes	Yes	0.00083
CH 012258	NPM1	NP_002511.1:p.Trp288CysfsTer12	NM_002520.6:c.860_863dup	0.0001	4	1	3	33661	6922	frameshift	5	170837543	C	CTCTG	Yes	Yes	0.00003
CH 012141	IDH2	NP_002159.2:p.Arg172Lys	NM_002168.3:c.515G>A	0.0038	61	32	36	13901	8377	missense	15	90631838	C	T	Yes	Yes	0.00474
CH 012347	IDH2	NP_002159.2:p.Arg140Gln	NM_002168.3:c.419G>A	0.0107	345	176	117	27465	16480	missense	15	90631934	C	T	Yes	Yes	0.01164
CH 012328	IDH2	NP_002159.2:p.Arg140Gln	NM_002168.3:c.419G>A	0.0303	450	286	141	14624	9446	missense	15	90631934	C	T	Yes	Yes	0.02849
CH 012362	IDH2	NP_002159.2:p.Arg140Gln	NM_002168.3:c.419G>A	0.0076	154	98	75	19977	12842	missense	15	90631934	C	T	Yes	Yes	0.00900
CH 012361	IDH2	NP_002159.2:p.Arg140Gln	NM_002168.3:c.419G>A	0.0437	684	434	188	15120	9927	missense	15	90631934	C	T	Yes	Yes	0.05181
CH 012361	NPM1	NP_002511.1:p.Trp288CysfsTer12	NM_002520.6:c.860_863dup	0.0007	15	4	9	21732	6226	frameshift	5	170837543	C	CTCTG	Yes	Yes	0.00073
CH 012437	IDH2	NP_002159.2:p.Arg140Gln	NM_002168.3:c.419G>A	0.0721	1530	911	302	21009	12641	missense	15	90631934	C	T	Yes	Yes	0.07500
CH 012465	IDH2	NP_002159.2:p.Arg140Gln	NM_002168.3:c.419G>A	0.343	7533	4243	531	21804	12370	missense	15	90631934	C	T	Yes	Yes	0.34390
CH 012481	IDH2	NP_002159.2:p.Arg140Gln	NM_002168.3:c.419G>A	0.0186	515	255	180	23955	13704	missense	15	90631934	C	T	Yes	Yes	0.02365
CH 016940	IDH2	NP_002159.2:p.Arg140Gln	NM_002168.3:c.419G>A	0.0279	640	385	211	22594	13812	missense	15	90631934	C	T	Yes	Yes	0.02673
CH 016868	IDH2	NP_002159.2:p.Arg140Gln	NM_002168.3:c.419G>A	0.0076	241	135	103	26455	17745	missense	15	90631934	C	T	Yes	Yes	0.01006
CH 016873	IDH2	NP_002159.2:p.Arg140Gln	NM_002168.3:c.419G>A	0.1872	4962	2373	443	26102	12676	missense	15	90631934	C	T	Yes	Yes	0.20223
CH 016952	IDH2	NP_002159.2:p.Arg140Gln	NM_002168.3:c.419G>A	0.0007	31	14	22	29891	20082	missense	15	90631934	C	T	Yes	Yes	0.00070
CH 016983	IDH2	NP_002159.2:p.Arg140Gln	NM_002168.3:c.419G>A	0.0045	111	63	67	22503	14083	missense	15	90631934	C	T	Yes	Yes	0.00531
CH 016833	IDH2	NP_002159.2:p.Arg140Gln	NM_002168.3:c.419G>A	0.2385	5958	3779	537	24744	15845	missense	15	90631934	C	T	Yes	Yes	0.24327
CH 016744	IDH2	NP_002159.2:p.Arg140Gln	NM_002168.3:c.419G>A	0.0106	172	115	80	15732	10836	missense	15	90631934	C	T	Yes	Yes	0.01303
CH 016557	IDH2	NP_002159.2:p.Arg172Lys	NM_002168.3:c.515G>A	0.0369	880	543	255	24362	14717	missense	15	90631838	C	T	Yes	Yes	0.04013
CH 016536	IDH2	NP_002159.2:p.Arg140Gln	NM_002168.3:c.419G>A	0.0043	95	59	54	19950	13857	missense	15	90631934	C	T	Yes	Yes	0.00416
CH 016988	IDH2	NP_002159.2:p.Arg140Gln	NM_002168.3:c.419G>A	0.0598	1219	757	283	19461	12651	missense	15	90631934	C	T	Yes	Yes	0.06244
CH 016515	IDH2	NP_002159.2:p.Arg140Gln	NM_002168.3:c.419G>A	0.0107	215	124	82	20010	11602	missense	15	90631934	C	T	Yes	Yes	0.01397
CH 016984	IDH2	NP_002159.2:p.Arg140Gln	NM_002168.3:c.419G>A	0.2775	4632	3195	478	16518	11515	missense	15	90631934	C	T	Yes	Yes	0.27775
CH 016629	IDH2	NP_002159.2:p.Arg140Gln	NM_002168.3:c.419G>A	0.0014	37	15	27	15900	10869	missense	15	90631934	C	T	Yes	Yes	0.00240
CH 016518	IDH2	NP_002159.2:p.Arg140Gln	NM_002168.3:c.419G>A	0.0303	436	285	140	13453	9414	missense	15	90631934	C	T	Yes	Yes	0.02724
CH 016553	IDH2	NP_002159.2:p.Arg140Gln	NM_002168.3:c.419G>A	0.0059	135	82	77	20332	13836	missense	15	90631934	C	T	Yes	Yes	0.00682
CH 016567	IDH2	NP_002159.2:p.Arg140Gln	NM_002168.3:c.419G>A	0.1912	3809	2452	463	19628	12822	missense	15	90631934	C	T	Yes	Yes	0.20364
CH 016660	IDH2	NP_002159.2:p.Arg140Gln	NM_002168.3:c.419G>A	0.0852	1153	779	269	13413	9144	missense	15	90631934	C	T	Yes	Yes	0.08690
CH 016624	IDH2	NP_002159.2:p.Arg140Gln	NM_002168.3:c.419G>A	0.0505	620	440	187	11965	8705	missense	15	90631934	C	T	Yes	Yes	0.05416
CH 016631	IDH2	NP_002159.2:p.Arg140Gln	NM_002168.3:c.419G>A	0.0015	31	15	22	13537	9764	missense	15	90631934	C	T	Yes	Yes	0.00248
CH 016712	IDH2	NP_002159.2:p.Arg140Gln	NM_002168.3:c.419G>A	0.2249	3081	2049	374	13518	9110	missense	15	90631934	C	T	Yes	Yes	0.22841
CH 016764	IDH2	NP_002159.2:p.Arg140Gln	NM_002168.3:c.419G>A	0.4032	6319	4380	463	15760	10862	missense	15	90631934	C	T	Yes	Yes	0.39821
CH 016702	IDH2	NP_002159.2:p.Arg140Gln	NM_002168.3:c.419G>A	0.01	162	104	81	15235	10380	missense	15	90631934	C	T	Yes	Yes	0.01291
CH 016550	IDH2	NP_002159.2:p.Arg140Gln	NM_002168.3:c.419G>A	0.0725	1579	1003	336	20198	13841	missense	15	90631934	C	T	Yes	Yes	0.08521
CH 016535	IDH2	NP_002159.2:p.Arg140Gln	NM_002168.3:c.419G>A	0.3385	7093	4806	488	20994	14200	missense	15	90631934	C	T	Yes	Yes	0.33460
CH 016535	NPM1	NP_002511.1:p.Trp288CysfsTer12	NM_002520.6:c.860_863dup	0.0003	10	0	5	38488	14072	frameshift	5	170837543	C	CTCTG	Yes	Yes	0.00014
CH 016951	IDH2	NP_002159.2:p.Arg140Gln	NM_002168.3:c.419G>A	0.2022	3479	2282	414	16914	11286	missense	15	90631934	C	T	Yes	Yes	0.20290
CH 016832	IDH2	NP_002159.2:p.Arg172Lys	NM_002168.3:c.515G>A	0.0063	159	93	77	25181	14854	missense	15	90631838	C	T	Yes	Yes	0.00772
CH 016751	IDH2	NP_002159.2:p.Arg172Lys	NM_002168.3:c.515G>A	0.0161	359	215	140	20370	13333	missense	15	90631838	C	T	Yes	Yes	0.01791
CH 016767	IDH2	NP_002159.2:p.Arg140Gln	NM_002168.3:c.419G>A	0.1334	1980	1267	359	14903	9499	missense	15	90631934	C	T	Yes	Yes	0.13745
CH 016724	IDH2	NP_002159.2:p.Arg140Gln	NM_002168.3:c.419G>A	0.4339	7310	4178	431	16908	9630	missense	15	90631934	C	T	Yes	Yes	0.42621
CH 016769	IDH2	NP_002159.2:p.Arg140Gln	NM_002168.3:c.419G>A	0.0321	612	411	188	18357	12805	missense	15	90631934	C	T	Yes	Yes	0.02933
CH 016755	IDH2	NP_002159.2:p.Arg140Gln	NM_002168.3:c.419G>A	0.0192	244	167	105	13044	8692	missense	15	90631934	C	T	Yes	Yes	0.01828
CH 016715	IDH2	NP_002159.2:p.Arg140Gln	NM_002168.3:c.419G>A	0.0234	371	226	118	15474	9648	missense	15	90631934	C	T	Yes	Yes	0.02335
CH 016757	IDH2	NP_002159.2:p.Arg140Gln	NM_002168.3:c.419G>A	0.301	6448	3738	492	21166	12419	missense	15	90631934	C	T	Yes	Yes	0.29870
CH 016664	IDH2	NP_002159.2:p.Arg140Trp	NM_002168.3:c.418C>T	0.478	7527	4682	505	15721	9796	missense	15	90631935	G	A	Yes	Yes	0.47839
CH 016678	IDH2	NP_002159.2:p.Arg140Gln	NM_002168.3:c.419G>A	0.0179	346	203	122	17453	11324	missense	15	90631934	C	T	Yes	Yes	0.01426
CH 016678	NPM1	NP_002511.1:p.Trp288CysfsTer12	NM_002520.6:c.863_864insCCTG	0.0137	410	82	159	29941	11736	frameshift	5	170837544	T	TCTGC	Yes	Yes	0.01639
CH 016461	IDH2	NP_002159.2:p.Arg140Gln	NM_002168.3:c.419G>A	0.0101	303	169	120	25232	16774	missense	15	90631934	C	T	Yes	Yes	0.01103
CH 016374	FLT3	NP_004110.2:p.Glu608_Asn609insTyrProThrAspSerLysTrpGluPheProArg	NM_004119.2:c.1824_1825insTATCCAACCGACTCCAAATGGGAGTTTCCAAGAGAA	0.0145	692		243	47856		FLT3-ITD	13	28608231	T	TTTCTCTTGGAAACTCCCATTTGGAGTCGGTTG GATA	Yes	Not Done	Not Done
CH 016374	IDH2	NP_002159.2:p.Arg140Gln	NM_002168.3:c.419G>A	0.0445	672	423	180	14478	9497	missense	15	90631934	C	T	Yes	Yes	0.04830
CH 016365	FLT3		NM_004119.2:c.1777_1837+2dup	0.004	443		191	109728		FLT3-ITD	13	28608216	T	TACCAAACTCTAAATTTTCTCTTGGAAACTCCCAT TTGAGATCATATTCATATTCTCTGAAATC	Yes	Not Done	Not Done
CH 016365	IDH2	NP_002159.2:p.Arg140Gln	NM_002168.3:c.419G>A	0.0536	1156	692	254	21720	12910	missense	15	90631934	C	T	Yes	Yes	0.05330
CH 016403	IDH2	NP_002159.2:p.Arg140Gln	NM_002168.3:c.419G>A	0.0095	147	103	67	16298	10850	missense	15	90631934	C	T	Yes	Yes	0.00738
CH 016487	IDH2	NP_002159.2:p.Arg172Lys	NM_002168.3:c.515G>A	0.0064	127	73	67	17827	11349	missense	15	90631838	C	T	Yes	Yes	0.00776
CH 016791	IDH2	NP_002159.2:p.Arg140Gln	NM_002168.3:c.419G>A	0.4153	5469	3827	498	13190	9216	missense	15	90631934	C	T	Yes	Yes	0.40851
CH 016787	IDH2	NP_002159.2:p.Arg140Gln	NM_002168.3:c.419G>A	0.1084	1791	1177	323	16266	10853	missense	15	90631934	C	T	Yes	Yes	0.11318
CH 016788	IDH2	NP_002159.2:p.Arg140Gln	NM_002168.3:c.419G>A	0.4059	7342	4870	561	17918	11997	missense	15	90631934	C	T	Yes	Yes	0.40696
CH 016415	FLT3	NP_004110.2:p.Tyr597_Glu598insAspGlyAsnGluTyrPheTyrValAspPheArgGluTyr	NM_004119.2:c.1793_1794insTGGTAATGAGTACTTCTACGTTGATTTCAGAGAATATGA	0.0015	100		53	64646		FLT3-ITD	13	28608262	T	TTCATATTCTCTGAAATCAACGTAGAAGTACTCAT TACCA	Yes	Not Done	Not Done
CH 016415	FLT3	NP_004110.2:p.Ser574_Phe594dup	NM_004119.2:c.1722_1784dup	0.0009	63		37	67940		FLT3-ITD	13	28608271	T	TCTGAAATCAACGTAGAAGTACTCATTATCTGAG GAGCCGGTCACCTGTACCATCTGTAGCTGG	Yes	Not Done	Not Done
CH 016415	IDH2	NP_002159.2:p.Arg140Gln	NM_002168.3:c.419G>A	0.383	7360	4550	506	19332	11879	missense	15	90631934	C	T	Yes	Yes	0.37251
CH 016545	FLT3	NP_004110.2:p.Arg607_Glu608insThrAspAsnGluTyrPheTyrValAspPheArgGluTyrGluTyrAspLeuLysTrpGluPheProArg	NM_004119.2:c.1821_1822insACAGATAATGAGTACTTCTACGTTGATTTCAGAGAATATGAATATGATCTCAAATGGGAGTTTCCAAGA	0.0016	95		57	59391		FLT3-ITD	13	28608234	C	CTCTTGGAAACTCCCATTTGAGATCATATTCATAT TCTCTGAAATCAACGTAGAAGTACTCATTATCTGT	Yes	Not Done	Not Done
CH 016545	IDH2	NP_002159.2:p.Arg140Gln	NM_002168.3:c.419G>A	0.0131	257	167	121	18340	12731	missense	15	90631934	C	T	Yes	Yes	0.01359
CH 016463	IDH2	NP_002159.2:p.Arg140Gln	NM_002168.3:c.419G>A	0.2314	4790	3076	447	20377	13292	missense	15	90631934	C	T	Yes	Yes	0.22202

Subject ID	Gene	HGVSp	HGVSc	VAF	Alternate Observations	Deep Alternate Observations	Unique Alternate Observations	Depth	Deep Depth	Variant Consequence	Chromosome	Position	Reference	Alternate	Previously reported*	ddPCR validated	ddPCR VAF
CH 016606	IDH2	NP_002159.2:p.Arg172Lys	NM_002168.3:c.515G>A	0.0034	83	43	54	19375	12574	missense	15	90631838	C	T	Yes	Yes	0.00507
CH 016604	FLT3	NP_004110.2:p.Leu610_Glu611insAsnValAspPheArgGluTyrGluTyrAspLeuLysTrpGluPheProArgGluAsnLeu	NM_004119.2:c.1830_1831insAACGTTGATTTCAGAGAATATGAATATGATCTCAAATGGGAGTTTCCAAGAGAAAATTTA	0.0014	152		105	109752		FLT3-ITD	13	28608225	C	CTAAATTTTCTCTTGGAAACTCCCATTTGAGATCATATTCATATTCTCTGAAATCAACGTT	Yes	Not Done	Not Done
CH 016604	IDH2	NP_002159.2:p.Arg140Gln	NM_002168.3:c.419G>A	0.0295	732	470	203	24045	15922	missense	15	90631934	C	T	Yes	Yes	0.03346
CH 016613	IDH2	NP_002159.2:p.Arg140Gln	NM_002168.3:c.419G>A	0.3757	7023	4765	518	18624	12683	missense	15	90631934	C	T	Yes	Yes	0.37459
CH 016650	IDH2	NP_002159.2:p.Arg140Gln	NM_002168.3:c.419G>A	0.3236	6062	4134	472	18676	12774	missense	15	90631934	C	T	Yes	Yes	0.31988
CH 016871	IDH2	NP_002159.2:p.Arg140Gln	NM_002168.3:c.419G>A	0.0145	427	237	166	26992	16330	missense	15	90631934	C	T	Yes	Yes	0.01772
CH 016808	IDH2	NP_002159.2:p.Arg140Gln	NM_002168.3:c.419G>A	0.0026	113	57	64	33136	22284	missense	15	90631934	C	T	Yes	Yes	0.00302
CH 016857	IDH2	NP_002159.2:p.Arg172Lys	NM_002168.3:c.515G>A	0.0007	30	13	25	35285	19504	missense	15	90631838	C	T	Yes	Yes	0.00063
CH 016843	IDH2	NP_002159.2:p.Arg140Gln	NM_002168.3:c.419G>A	0.0188	616	345	190	29448	18351	missense	15	90631934	C	T	Yes	Yes	0.02560
CH 016582	IDH2	NP_002159.2:p.Arg140Gln	NM_002168.3:c.419G>A	0.001	58	19	35	31330	19911	missense	15	90631934	C	T	Yes	Yes	0.00131
CH 016949	IDH2	NP_002159.2:p.Arg140Gln	NM_002168.3:c.419G>A	0.0735	2284	1388	377	29986	18883	missense	15	90631934	C	T	Yes	Yes	0.08409
CH 017037	FLT3	NP_004110.2:p.Asp586_Pro606dup	NM_004119.2:c.1756_1818dup	0.0006	62		37	104591		FLT3-ITD	13	28608237	T	TTGGAAACTCCCATTTGAGATCATATTCATATTCT	Yes	Not Done	Not Done
CH 017037	NPM1	NP_002511.1:p.Trp288CysfsTer12	NM_002520.6:c.860_863dup	0.0005	24	3	13	44978	15236	frameshift	5	170837543	C	CTCTG	Yes	Yes	0.00057
CH 016892	IDH2	NP_002159.2:p.Arg140Gln	NM_002168.3:c.419G>A	0.147	4728	2945	507	31527	20038	missense	15	90631934	C	T	Yes	Yes	0.16445
CH 016858	IDH2	NP_002159.2:p.Arg140Gln	NM_002168.3:c.419G>A	0.014	547	272	178	33308	19462	missense	15	90631934	C	T	Yes	Yes	0.01958
CH 016924	IDH2	P_002159.2:p.Arg172_His173delinsSer	NM_002168.3:c.516_518inv	0.0503	1517	965	342	29537	19197	missense	15	90631835	TGC	GCA	Yes	Not Done	Not Done
CH 016924	NPM1	NP_002511.1:p.Trp288CysfsTer12	NM_002520.6:c.860_863dup	0.0004	17	3	10	42159	14323	frameshift	5	170837543	C	CTCTG	Yes	Yes	0.00043
CH 016882	IDH2	NP_002159.2:p.Arg140Gln	NM_002168.3:c.419G>A	0.2734	4766	3238	485	17605	11843	missense	15	90631934	C	T	Yes	Yes	0.27170
CH 016916	IDH2	NP_002159.2:p.Arg140Gln	NM_002168.3:c.419G>A	0.1799	4021	2691	495	22039	14960	missense	15	90631934	C	T	Yes	Yes	0.18173
CH 016856	FLT3	NP_004110.2:p.Phe594_Asn609dup	NM_004119.2:c.1782_1829dup	0.0159	1612		382	101434		FLT3-ITD	13	28608226	T	TAAATTTTCTCTTGGAAACTCCCATTTGAGATCAT	Yes	Not Done	Not Done
CH 016856	IDH2	NP_002159.2:p.Arg140Gln	NM_002168.3:c.419G>A	0.0131	412	240	148	28495	18376	missense	15	90631934	C	T	Yes	Yes	0.01695
CH 016856	NPM1	NP_002511.1:p.Trp288CysfsTer12	NM_002520.6:c.863_864insCCTG	0.0111	402	47	138	36207	8685	frameshift	5	170837544	T	TCTGC	Yes	Yes	0.01413
CH 017057	IDH2	NP_002159.2:p.Arg140Gln	NM_002168.3:c.419G>A	0.0165	315	196	128	17778	11895	missense	15	90631934	C	T	Yes	Yes	0.01749
CH 017057	NPM1	NP_002511.1:p.Trp288CysfsTer12	NM_002520.6:c.860_863dup	0.0001	3	1	2	26673	9008	frameshift	5	170837543	C	CTCTG	Yes	No	0
CH 016903	IDH2	NP_002159.2:p.Arg140Gln	NM_002168.3:c.419G>A	0.0026	79	35	35	20756	13367	missense	15	90631934	C	T	Yes	Yes	0.00313
CH 016919	FLT3	NP_004110.2:p.Tyr599_Asp600insSerProThrGlySerSerAspAsnGluTyrPheTyrValAspPheArgGluTyrGluTyr	NM_004119.2:c.1797_1798insTCCCCGACCGGCTCCTCAGATAATGAGTACTTCTACGTTGATTTCAGAGAATATGAATAT	0.0002	11		7	62140		FLT3-ITD	13	28608258	C	CATATTCATATTCTCTGAAATCAACGTAGAAGTACTCATTATCTGAGGAGCCGGTCGGGGA	Yes	Not Done	Not Done
CH 016919	IDH2	NP_002159.2:p.Arg140Gln	NM_002168.3:c.419G>A	0.0254	526	325	170	19894	12812	missense	15	90631934	C	T	Yes	Yes	0.02890
CH 016937	IDH2	NP_002159.2:p.Arg140Gln	NM_002168.3:c.419G>A	0.0011	29	9	19	15569	8043	missense	15	90631934	C	T	Yes	Yes	0.00100
CH 016964	IDH2	NP_002159.2:p.Arg140Gln	NM_002168.3:c.419G>A	0.0028	40	27	26	13565	9511	missense	15	90631934	C	T	Yes	Not Done	Not Done
CH 016962	IDH2	NP_002159.2:p.Arg140Gln	NM_002168.3:c.419G>A	0.2925	4087	2783	437	13819	9515	missense	15	90631934	C	T	Yes	Yes	0.28943
CH 017026	IDH2	NP_002159.2:p.Arg140Gln	NM_002168.3:c.419G>A	0.0816	1770	1163	311	21411	14252	missense	15	90631934	C	T	Yes	Yes	0.08612
CH 017003	FLT3	NP_004110.2:p.Asn587_Asp600dup	NM_004119.2:c.1759_1800dup	0.0107	686		159	64131		FLT3-ITD	13	28608255	G	GATCATATTCATATTCTCTGAAATCAACGTAGAAG	Yes	Not Done	Not Done
CH 017003	IDH2	NP_002159.2:p.Arg140Gln	NM_002168.3:c.419G>A	0.0041	94	47	61	19298	11513	missense	15	90631934	C	T	Yes	Yes	0.00614
CH 017104	IDH2	NP_002159.2:p.Arg140Gln	NM_002168.3:c.419G>A	0.1216	3209	1952	394	25304	16049	missense	15	90631934	C	T	Yes	Yes	0.12794
CH 017059	IDH2	NP_002159.2:p.Arg140Gln	NM_002168.3:c.419G>A	0.0023	67	34	41	22900	14572	missense	15	90631934	C	T	Yes	Yes	0.00397
CH 017116	IDH2	NP_002159.2:p.Arg140Gln	NM_002168.3:c.419G>A	0.0058	123	76	61	20693	13214	missense	15	90631934	C	T	Yes	Yes	0.00577
CH 017054	IDH2	NP_002159.2:p.Arg172Lys	NM_002168.3:c.515G>A	0.0808	1707	1107	319	20929	13695	missense	15	90631838	C	T	Yes	Yes	0.09242
CH 017093	IDH2	NP_002159.2:p.Arg140Gln	NM_002168.3:c.419G>A	0.0223	676	385	209	28982	17272	missense	15	90631934	C	T	Yes	Yes	0.02612
CH 017370	IDH2	NP_002159.2:p.Arg140Gln	NM_002168.3:c.419G>A	0.2527	5827	3172	573	22565	12553	missense	15	90631934	C	T	No	Yes	0.2743
CH 017371	IDH2	NP_002159.2:p.Arg140Gln	NM_002168.3:c.419G>A	0.1167	2955	1865	424	24991	15984	missense	15	90631934	C	T	No	Yes	0.1255
CH 017401	IDH2	NP_002159.2:p.Arg172Lys	NM_002168.3:c.515G>A	0.0005	25	9	20	27646	17481	missense	15	90631838	C	T	No	Yes	0.0012
CH 017407	IDH2	NP_002159.2:p.Arg140Gln	NM_002168.3:c.419G>A	0.5626	16165	9944	741	29000	17676	missense	15	90631934	C	T	No	Yes	0.5595
CH 017414	IDH2	NP_002159.2:p.Arg140Gln	NM_002168.3:c.419G>A	0.3065	6665	3869	539	21240	12624	missense	15	90631934	C	T	No	Yes	0.32
CH 017417	FLT3	NP_004110.2:p.Glu598_Tyr599insHisValAspPheArgGluTyrGlu	NM_004119.2:c.1793_1794insGCACGTTGATTTCAGAGAATATGA	0.0002	13		10	78663		FLT3-ITD	13	28608262	T	TTCATATTCTCTGAAATCAACGTGC	No	Not Done	Not Done
CH 017417	IDH2	NP_002159.2:p.Arg140Gln	NM_002168.3:c.419G>A	0.0022	58	30	36	21510	13572	missense	15	90631934	C	T	No	Yes	0.0031
CH 017422	IDH2	NP_002159.2:p.Arg140Gln	NM_002168.3:c.419G>A	0.0508	886	600	228	17303	11821	missense	15	90631934	C	T	No	Yes	0.0529
CH 017424	IDH2	NP_002159.2:p.Arg172Lys	NM_002168.3:c.515G>A	0.0024	66	27	45	21550	11199	missense	15	90631838	C	T	No	Yes	0.0041
CH 017425	FLT3	NP_004110.2:p.Gly583_Leu601dup	NM_004119.2:c.1747_1803dup	0.0118	576		63	48885		FLT3-ITD	13	28608253	G	GAGATCATATTCATATTCTCTGAAATCAACGTAGA	No	Not Done	Not Done
CH 017425	NPM1	NP_002511.1:p.Trp288CysfsTer12	NM_002520.6:c.860_863dup	0.0014	24	4	17	16781	2673	frameshift	5	170837543	C	CTCTG	No	Not Done	Not Done
CH 017427	IDH2	NP_002159.2:p.Arg172Lys	NM_002168.3:c.515G>A	0.0015	46	16	28	26512	10872	missense	15	90631838	C	T	No	Yes	0.002
CH 017450	IDH2	NP_002159.2:p.Arg140Gln	NM_002168.3:c.419G>A	0.2597	6247	3832	505	23758	14755	missense	15	90631934	C	T	No	Yes	0.2743
CH 017457	IDH2	NP_002159.2:p.Arg140Trp	NM_002168.3:c.418C>T	0.0022	59	28	43	20919	12575	missense	15	90631935	G	A	No	Yes	0.0022
CH 017460	FLT3	NP_004110.2:p.Tyr597_Glu608dup	NM_004119.2:c.1789_1824dup	0.0002	17		15	85222		FLT3-ITD	13	28608237	T	TTGGAAACTCCCATTTGAGATCATATTCATATTCT	No	Not Done	Not Done
CH 017460	IDH2	NP_002159.2:p.Arg140Gln	NM_002168.3:c.419G>A	0.0029	73	41	39	20356	14248	missense	15	90631934	C	T	No	Yes	0.0028
CH 017476	IDH2	NP_002159.2:p.Arg140Gln	NM_002168.3:c.419G>A	0.1767	3621	2126	416	19873	12034	missense	15	90631934	C	T	No	Yes	0.1927
CH 017478	IDH2	NP_002159.2:p.Arg140Gln	NM_002168.3:c.419G>A	0.0132	318	192	140	22954	14586	missense	15	90631934	C	T	No	Yes	0.0122
CH 017482	IDH2	NP_002159.2:p.Arg140Gln	NM_002168.3:c.419G>A	0.022	472	287	166	20785	13042	missense	15	90631934	C	T	No	Yes	0.0222
CH 017489	IDH2	NP_002159.2:p.Arg140Gln	NM_002168.3:c.419G>A	0.234	4236	2425	445	18113	10362	missense	15	90631934	C	T	No	Yes	0.2393
CH 017522	IDH2	NP_002159.2:p.Arg140Gln	NM_002168.3:c.419G>A	0.0891	1701	1102	345	18758	12372	missense	15	90631934	C	T	No	Yes	0.0959
CH 017528	IDH2	NP_002159.2:p.Arg172Lys	NM_002168.3:c.515G>A	0.1638	3788	1391	413	22389	8494	missense	15	90631838	C	T	No	Yes	0.1874
CH 017536	IDH2	NP_002159.2:p.Arg140Gln	NM_002168.3:c.419G>A	0.2982	5695	2966	458	18666	9947	missense	15	90631934	C	T	No	Yes	0.3085
CH 017572	IDH2	NP_002159.2:p.Arg140Gln	NM_002168.3:c.419G>A	0.0749	1387	875	293	17485	11684	missense	15	90631934	C	T	No	Yes	0.0818
CH 017574	IDH2	NP_002159.2:p.Arg140Gln	NM_002168.3:c.419G>A	0.1639	3141	2128	402	19092	12980	missense	15	90631934	C	T	No	Yes	0.1697
CH 017600	IDH2	NP_002159.2:p.Arg140Gly	NM_002168.3:c.418C>G	0.0018	45	25	29	20433	13587	missense	15	90631935	G	C	No	Not Done	Not Done
CH 017605	IDH2	NP_002159.2:p.Arg140Gln	NM_002168.3:c.419G>A	0.0014	31	18	21	18753	12815	missense	15	90631934	C	T	No	Yes	0.0018
CH 017623	IDH2	NP_002159.2:p.Arg140Gln	NM_002168.3:c.419G>A	0.3129	5654	3781	464	17989	12083	missense	15	90631934	C	T	No	Yes	0.3171
CH 017635	IDH2	NP_002159.2:p.Arg140Gln	NM_002168.3:c.419G>A	0.0019	53	27	33	21600	14000	missense	15	90631934	C	T	No	Yes	0.0035
CH 017637	IDH2	NP_002159.2:p.Arg140Gln	NM_002168.3:c.419G>A	0.0136	234	164	114	18008	12025	missense	15	90631934	C	T	No	Yes	0.0168
CH 017639	IDH2	NP_002159.2:p.Arg172Lys	NM_002168.3:c.515G>A	0.0011	35	12	29	27029	11255	missense	15	90631838	C	T	No	Yes	0.0013
CH 017656	IDH2	NP_002159.2:p.Arg140Gln	NM_002168.3:c.419G>A	0.0094	207	127	97	21124	13507	missense	15	90631934	C	T	No	Yes	0.0097
CH 017661	IDH2	NP_002159.2:p.Arg140Trp	NM_002168.3:c.418C>T	0.0085	264	132	115	28871	15556	missense	15	90631935	G	A	No	Yes	0.0086
CH 017666	IDH2	NP_002159.2:p.Arg140Gln	NM_002168.3:c.419G>A	0.1023	2170	1441	332	20969	14091	missense	15	90631934	C	T	No	Yes	0.1153
